# M3S-GRPred: a novel ensemble learning approach for the interpretable prediction of glucocorticoid receptor antagonists using a multi-step stacking strategy

**DOI:** 10.1186/s12859-025-06132-1

**Published:** 2025-04-30

**Authors:** Nalini Schaduangrat, Hathaichanok Chuntakaruk, Thanyada Rungrotmongkol, Pakpoom Mookdarsanit, Watshara Shoombuatong

**Affiliations:** 1 Faculty of Medical Technology, Center for Research Innovation and Biomedical Informatics, Mahidol University, Bangkok, 10700 Thailand; 2 Program in Bioinformatics and Computational Biology, Graduate School, Chulalongkorn University, Bangkok, 10330 Thailand; 3 Faculty of Science, Center of Excellence in Structural and Computational Biology, Chulalongkorn University, Bangkok, 10330 Thailand; 4 Faculty of Medicine, Center for Artificial Intelligence in Medicine, Chulalongkorn University, Bangkok, Bangkok, 10330 Thailand; 5 Faculty of Science, Computer Science and Artificial Intelligence, Chandrakasem Rajabhat University, Bangkok, 10900 Thailand

**Keywords:** Cushing’s syndrome, Glucocorticoid receptor, QSAR, Cheminformatics, Machine learning, Feature selection, Multi-view feature

## Abstract

Accelerating drug discovery for glucocorticoid receptor (GR)-related disorders, including innovative machine learning (ML)-based approaches, holds promise in advancing therapeutic development, optimizing treatment efficacy, and mitigating adverse effects. While experimental methods can accurately identify GR antagonists, they are often not cost-effective for large-scale drug discovery. Thus, computational approaches leveraging SMILES information for precise in silico identification of GR antagonists are crucial, enabling efficient and scalable drug discovery. Here, we develop a new ensemble learning approach using a multi-step stacking strategy (M3S), termed M3S-GRPred, aimed at rapidly and accurately discovering novel GR antagonists. To the best of our knowledge, M3S-GRPred is the first SMILES-based predictor designed to identify GR antagonists without the use of 3D structural information. In M3S-GRPred, we first constructed different balanced subsets using an under-sampling approach. Using these balanced subsets, we explored and evaluated heterogeneous base-classifiers trained with a variety of SMILES-based feature descriptors coupled with popular ML algorithms. Finally, M3S-GRPred was constructed by integrating probabilistic feature from the selected base-classifiers derived from a two-step feature selection technique. Our comparative experiments demonstrate that M3S-GRPred can precisely identify GR antagonists and effectively address the imbalanced dataset. Compared to traditional ML classifiers, M3S-GRPred attained superior performance in terms of both the training and independent test datasets. Additionally, M3S-GRPred was applied to identify potential GR antagonists among FDA-approved drugs confirmed through molecular docking, followed by detailed MD simulation studies for drug repurposing in Cushing’s syndrome. We anticipate that M3S-GRPred will serve as an efficient screening tool for discovering novel GR antagonists from vast libraries of unknown compounds in a cost-effective manner.

**Supplementary Information:**

The online version contains supplementary material available at 10.1186/s12859-025-06132-1.

## Introduction

The glucocorticoid receptor (GR), a widely expressed transcription factor activated by ligands and part of the nuclear receptor superfamily, mediates various biological processes. These processes include gluconeogenesis, inflammation, immunity, bone metabolism, cardiovascular function, overall homeostasis and development, and brain function [1]. It is essential for survival, as mice with a disrupted GR cannot survive postpartum due to multiple defects [2]. Cortisol, the natural hormone that binds to GR, is secreted by the adrenal glands and regulated by adrenocorticotropic hormone (ACTH). Cushing syndrome results from excessive glucocorticoid exposure, causing significant morbidity and mortality. It can develop from corticosteroid administration (exogenous) or uncontrolled cortisol hypersecretion, whether ACTH-dependent or independent (endogenous) [3, 4]. Presently, there are two medical treatments for endogenous Cushing’s syndrome that have received approval from the US Food and Drug Administration (FDA). The first approved medical therapy is mifepristone, a nonselective GR antagonist, designated for adult patients with glucose intolerance or type 2 diabetes mellitus coupled with Cushing’s syndrome and are either ineligible for surgery or have undergone unsuccessful surgery [5]. The second approved medical therapy is pasireotide, characterized as an agonist of the somatostatin receptor. It is approved specifically for those diagnosed with a subset of Cushing’s syndrome called Cushing’s disease, when pituitary surgery is not a viable option or has proven ineffective [6].

As previously mentioned, Cushing’s syndrome is caused by excessive cortisol activity, leading to severe symptoms like excess trunk fat, thin arms and legs, rounded face, and a fatty hump between the shoulders. Patients often experience diabetes, hypertension, skin issues, and psychiatric disturbances [7, 8]. Moreover, elevated cortisol levels are linked to a heightened risk of cardiovascular events such as myocardial infarction, cerebrovascular events like sepsis, thromboembolism, and stroke, leading to a greater mortality risk compared to the general population [9–11]. Mifepristone effectively alleviates the clinical effects of elevated cortisol by acting as a GR antagonist, improving patients’ overall condition [5]. However, it does not reduce cortisol production and has drawbacks due to its non-selectivity. Its strong affinity for the progesterone receptor (PR) can cause pregnancy termination and issues like irregular vaginal bleeding or endometrial thickening in some patients [12]. Currently, a Phase 3 clinical trial for GR antagonist, Relacorilant is under evaluation (NCT02804750). Consequently, there is an ongoing need to discover new GR antagonists with diverse properties suitable for therapeutic purposes in various diseases involving GR signaling. However, the conventional drug discovery process is known for being prolonged and time-intensive. To expedite this process, machine learning (ML)-based approaches have proven to be highly effective. Moreover, researchers are exploring diverse computer-assisted approaches for GR drug design, including the prediction of quantitative structure–activity relationship (QSAR) for GR using models based on ML [13–16], deep learning [17, 18], molecular docking [19–22], molecular dynamic simulations [23–26], and pharmacophore analysis [25, 27, 28], among others.

Here, we present a novel stacked ensemble learning approach, named M3S-GRPred, designed to rapidly and accurately discover novel GR antagonists. The major contributions of the proposed strategy can be summarized as follows: (i) We developed a multi-step stacking strategy (M3S) to develop M3S-GRPred for solving the data imbalance problem (i.e., 1,314 active compounds and 275 inactive compounds). Unlike the conventional stacking strategy, the M3S-GRPred method employed an under-sampling approach to construct different balanced subsets. Using these balanced subsets, we evaluated and compared the performance of various base-classifiers trained with five SMILES-based feature descriptors (i.e., AP2DC, CDKExt, FP4C, MACCS, and Pubchem) coupled with popular ML algorithms (i.e., KNN, MLP, PLS, RF, SVM, and XGB). All the base-classifiers were employed to generate probabilistic features (PFs) based on the ten-fold cross-validation procedure. Finally, we utilized a two-step feature selection to optimize these PFs and determine the best feature subset for constructing the final ensemble learning model using stacking strategy; (ii) M3S-GRPred is the first SMILES-based stacked model for the identification of GR antagonists; (iii) Experimental results showed that the M3S method not only addresses the data imbalance problem, but also achieves more stable and accurate identification of GR antagonists. Specifically, as indicated by the independent test, M3S-GRPred outperformed several traditional ML classifiers, achieving a balanced accuracy (BACC) of 0.891, Matthews correlation coefficient (MCC) of 0.658 and, area under the receiver-operating curves (AUC) of 0.953; and (iv) The proposed M3S-GRPred was applied to identify important features for GR antagonists and to determine FDA-approved drugs that could potentially act as GR antagonists using molecular docking and MD simulation studies. As a result, M3S-GRPred identified two FDA-approved drugs as potential GR antagonists.

## Materials and methods

### Training and independent test datasets

In this study, compounds were sourced from the ChEMBL database (Target: GR; ID: CHEMBL2034) [29]. Initially, 13,227 compounds exhibiting activity towards GR were retrieved and subjected to data curation using our in-house code in the R programming environment [30]. During this process, compounds with ‘=’ symbols in their “Standard.Value” column were retained, while those with symbols such as ‘<’, ‘>’, or ‘/’ were excluded. Redundant and missing data points were also eliminated. Subsequently, the dataset was refined by selecting compounds with quantitative IC_50_ bioactivity values obtained from functional and cell-based assays relevant to GR activity, resulting in a final dataset of 1632 compounds. To enhance data clarity and enable comparisons of drug potency at the equimolar concentrations, these compounds were converted into their pIC_50_ values (negative logarithm base 10 of IC_50_ in Molar concentration) and further processed according to our previous works [31–34]. Consequently, the final dataset comprised 1314 active compounds and 275 inactive compounds. Among these, we applied 75% and 25% of all the active and inactive compounds to construct the training and independent test datasets, respectively.

### Feature extraction methods

Feature extraction involves characterizing molecules of interest through quantitative and qualitative descriptors that encompass their structural composition, connectivity, and physicochemical traits [28]. In our study, we implemented data preprocessing steps using the PADEL-descriptor software [35], which included eliminating salts, removing duplicate data, and standardizing tautomers. Following this preprocessing phase, we utilized the SMILES notation of the compounds under analysis to generate molecular fingerprints. A total of five distinct fingerprint types were employed in this research, including AP2DC, CDKExt, FP4C, MACCS, and Pubchem [36–40]. For a detailed description of each fingerprint, please refer to Table 1. All calculations related to molecular fingerprint generation were conducted within the R [30] programming environment.

**Table 1:** Summary of five SMILES-based feature descriptors used in this study

Fingerprint	#Feature	Description	References
AP2D	780	Presence of atom pairs at various topological distances	[36]
CKDExt	1024	Extends the fingerprint with additional bits describing ring features	[40]
MACCS	166	Binary representation of chemical features defined by MACCS keys	[37]
Pubchem	881	Binary representation of substructures defined by PubChem	[38]
FP4C	307	Count of SMARTS patterns for functional groups	[39]

### Overall framework of M3S-GRPred

**Fig. 1:**
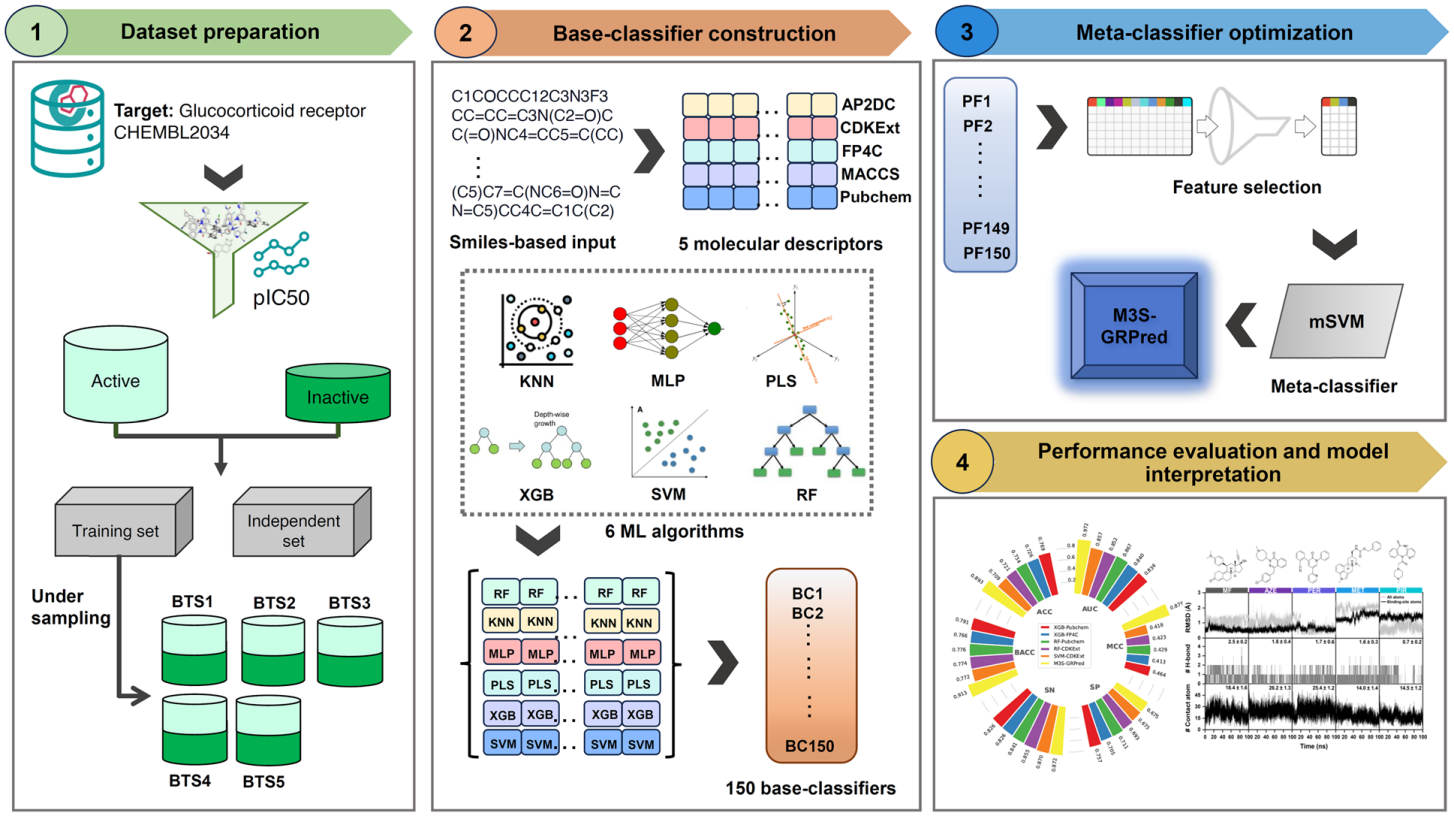
System flowchart of the proposed M3S-GRPred. The overall workflow for the development of M3S-GRPred contains three major steps: preparing balanced training subsets, constructing base-classifiers, optimizing meta-classifiers, performance evaluation and model interpretation

M3S is a novel multi-step stacking strategy, which is developed for solving the data imbalance issue by leveraging the stacking strategy coupled with the under-sampling technique [41, 42]. Recently, this approach was successfully applied to the interpretable identification of IL-6 inducing peptides [41] and allergenicity of chemical compounds [42]. Here, we applied the M3S strategy for discovering novel GR antagonists. The overall framework of M3S-GRPred is divided into the following steps (Fig. 1), which include: (i) preparing the balanced training datasets; (ii) constructing base-classifiers; and (iii) optimizing meta-classifiers.

In the first step, balanced training subsets (BTS) were established using the under-sampling approach on the original training dataset, which initially contained 790 positives and 159 negatives. Given the 5:1 ratio between positives and negatives, we under-sampled from the positive samples five times to create five balanced training subsets (i.e., BTS1–BTS5). Importantly, there is no overlap among these five positive subsets. Utilizing all five positive subsets allows us to maximize the utility of the information provided by the active compounds. In the second step, for each balanced training subset, 30 base-classifier were constructed based on six ML algorithms (i.e., KNN, MLP, PLS, RF, SVM, and XGB) in conjunction with five molecular descriptors (i.e., AP2DC, CDKExt, FP4C, MACCS, and Pubchem). These six ML algorithms are widely applied in research related to drug discovery and development [43–45]. By utilizing all balanced training subsets, we obtained a total of 150 well-trained base-classifiers. All base-classifiers were built and optimized using the caret package in the R programming environment [46], with optimal parameters determined through ten-fold cross-validation (Supplementary Table S2).

At the final step, a probability feature vector (PFV) was established using probability scores from the 150 base-classifiers for being GR antagonists [41, 47–49]. For a given compound *C*, its PFV can be represented by:

1 $$\text{PFV}=\left[{\text{P}(\text{ML}}_{1}, {\text{MD}}_{1},{\text{BTS}}_{1}\right),{\text{P}(\text{ML}}_{1}, {\text{MD}}_{1},{\text{BTS}}_{2}),\dots .,{\text{P}(\text{ML}}_{6}, {\text{MD}}_{5},{\text{BTS}}_{5})]$$

where ${\text{P}(\text{ML}}_{\text{i}}, {\text{MD}}_{\text{j}},{\text{BTS}}_{k}),$ denotes the probability score derived from the base-classifier trained with the *i*th ML algorithm and the *j*th feature encoding over the *k*th balanced training subset. Thus, the PFV of the compound *C* could be represented by a 150-D probabilistic feature vector. To enhance the learning accuracy, a two-step feature selection strategy was employed to determine the best feature subset containing *m* useful PFs to develop the proposed model. The two-step feature selection implementation herein is the same as applied in our previous studies [45, 50, 51]. In this strategy, all PFs were initially ranked based on their RF-based mean decrease of Gini index (MDGI). Fifteen feature subsets containing *m* top-ranked importance PFs were generated, where *m* = 10, 20, 30,…, 150. Subsequently, each feature subset was used to train 15 different SVM-based meta-classifiers independently. The feature subset yielding the highest cross-validation Matthews Correlation Coefficient (MCC) was selected as the best feature subset.

### Performance evaluation

Herein, we employed two standard evaluation strategies to assess the robustness and generalization ability of the prediction models, including ten-fold cross-validation and independent tests. In the meanwhile, six performance measures: MCC, ACC, AUC, sensitivity (SN), and specificity (SP) were used to evaluate the performance of the prediction models [52, 53]. These performance measures are defined as

2 $$\text{SN}=\frac{\text{TP}}{\left(\text{TP}+\text{FN}\right)}$$

3 $$\text{SP}=\frac{\text{TN}}{\left(\text{TN}+\text{FP}\right)}$$

4 $$\text{ACC}=\frac{\text{TP}+\text{TN}}{\left(\text{TP}+\text{TN}+\text{FP}+\text{FN}\right)}$$

5 $$\text{MCC}=\frac{\text{TP}\times \text{TN}-\text{FP}\times \text{FN}}{\sqrt[]{(\text{TP}+\text{FP})(\text{TP}+\text{FN})(\text{TN}+\text{FP})(\text{TN}+\text{FN})}}$$

6 $$\text{BACC}=(\text{SN}+\text{SP})/2$$

where the numbers of correctly predicted positive and negative samples were referred to as TP and TN, respectively. On the other hand, the numbers of falsely predicted positive and negative samples are referred to as FP and FN, respectively [41, 42, 47, 49, 54, 55].

### Molecular docking study of FDA-approved drugs

A library of 2735 FDA-approved small molecule drugs was obtained from the DrugBank database (version 5.1.10; released on January 4, 2023). After eliminating inorganic compounds, salt, SMILES with explicit valence, disconnected SMILES representations, and duplicates, the number of compounds was reduced to 1737. Molecular descriptors were computed for these 1737 compounds, and used as input for predictions with our model. To aid in drug repurposing efforts, the top 30 compounds identified by our model were then subjected to docking analysis using the 3D structure of GR (PDB ID: 1NHZ) obtained from the Protein Data Bank (https://www.rcsb.org/) and prepared for docking in UCSF Chimera X [56]. The refined structure underwent energy optimization and minimization using the OPLS3 force field with 5000 steps of steepest descent (step size = 0.02 Å) and 500 steps of conjugate gradient (step size = 0.02 Å). A receptor grid box was constructed using MGLTools 1.5.7 [57], with dimensions X = 56, Y = 64, and Z = 62, centered at coordinates − 5.31 Å, 14.112 Å, and 5.61 Å based on active site residues within the GR binding pocket. This coordinate space was utilized as the docking site. To validate the docking approach, the co-crystallized ligand (mifepristone; RU486) in the crystal structure was re-docked into the active site of GR to assess the ability of the docking method to replicate the native conformation of the inhibitor. The docking run employed 20 binding modes, an energy range of 4, and an exhaustiveness of 32. Docked complexes were visualized using PyMOL [58].

### Molecular dynamics (MD) simulations

The docked conformation of four screened compounds (i.e., azelastine (AZE), metergoline (MET), perampanel (PER), and pirenzepine (PIR)) bound to the GR, along with the GR/MF (Mifepristone) crystal structure [59], were used as initial structures for MD simulations using AMBER22 [60] with periodic conditions as detailed in previous studies [24, 61–63]. The protonation state of protein receptors was determined using the PROPKA in the PDB2PQR webserver [64]. The electrostatic potential (ESP) charges for optimized drugs were derived at the HF/6-31(d) theory level and incorporated into restrained ESP (RESP) charges through the ANTECHAMBER module of AmberTools21 [60]. The AMBER force fields ff19SB and GAFF2 were applied for the protein and drug, respectively. Missing hydrogen atoms were added using the tLEaP module. The system was neutralized with counterions and immersed in the TIP3P water model in the octahedral box [65] extended at least 10 Å from the protein surface. Structural minimization was performed with 1500 steps of steepest descent (SD) followed by conjugated gradient (CG) methods. Subsequently, MD simulations with a 2-fs time step were executed, with nonbonded interactions limited of 10 Å, and long-range electrostatic interactions [66] treated using the Particle Mesh Ewald (PME) summation approach. Pressure and temperature were controlled, and covalent bonds involving hydrogen atoms were constrained using the SHAKE methodology [67]. The models were heated from 10 to 310 K for 100 ps and maintained at 310 K for 100 ns, with three replicates using different velocities. The CPPTRAJ module was used to analyze all-atom root mean square deviation (RMSD), intermolecular hydrogen bonds, and contact atoms between the drug and protein over the production phase [68]. Binding affinity of the drug/protein complex for each simulation was estimated using Molecular mechanics with the Generalized-Born (MM/GBSA) [69] or Poisson-Boltzmann (MM/PBSA) [70] surface area solvation calculations, using 100 snapshots from the last 20 ns. Drug-ligand interactions were. Visualized with LigandScout 4.4.8 [71].

## Results and discussion

### Chemical space analysis

**Fig. 2:**
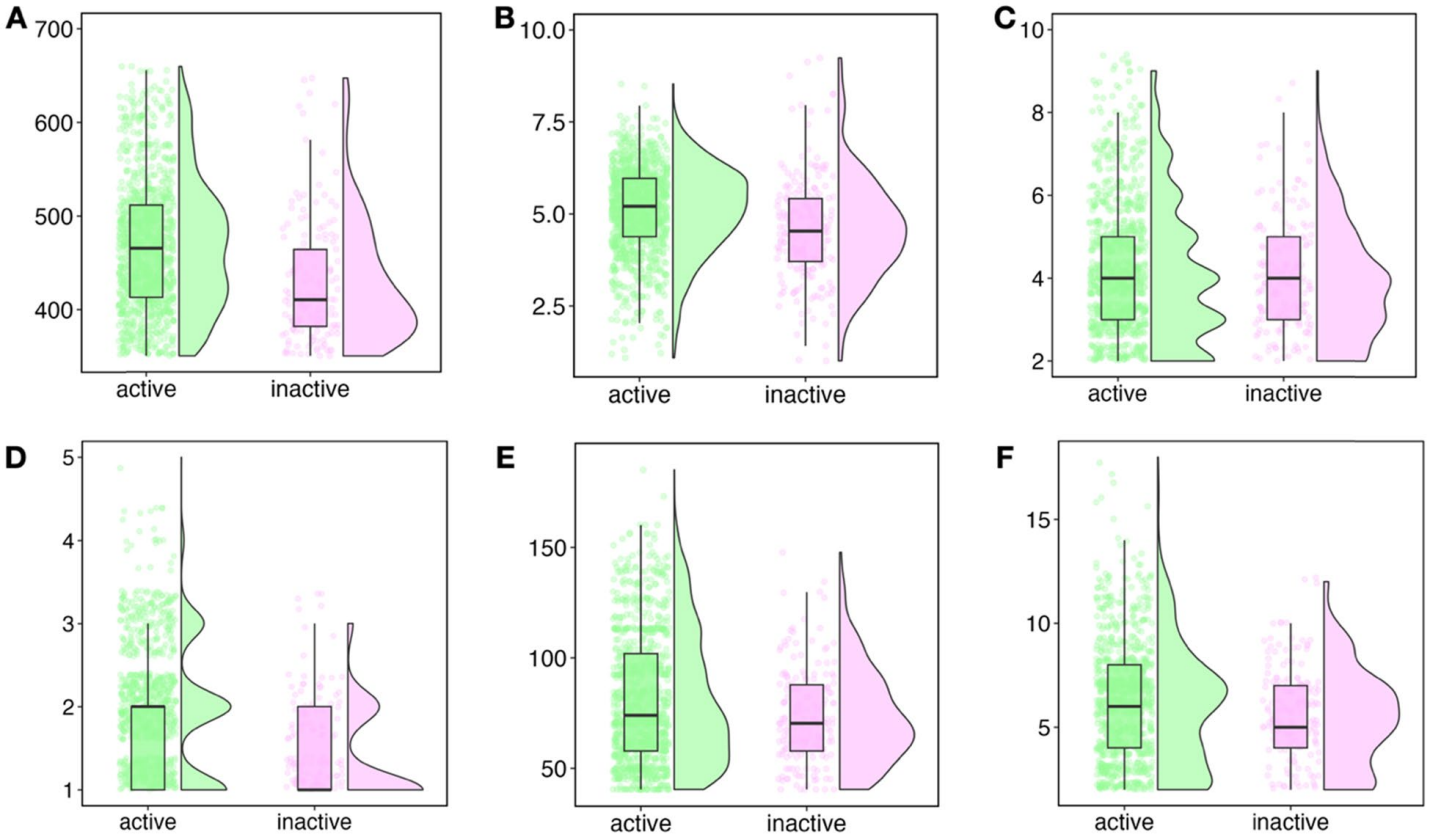
Plot of physicochemical properties where **A** MW, **B** AlogP, **C** HBA, **D** HBD, **E** TPSA, and **F** nRotB represent the chemical space of the dataset as visualized through the two classes of compounds (i.e., active and inactive) represented by green and pink colours, respectively

Chemical space analysis in drug discovery aims to understand the distribution of chemical compounds, categorized as active and inactive, based on their physicochemical properties. This involves examining various descriptors such as molecular weight (MW), octanol–water partition coefficient (AlogP), hydrogen bond acceptor count (HBA), hydrogen bond donor count (HBD), topological polar surface area (TPSA), and rotatable bond count (nRotB). Lipinski’s Rule of Five (Ro5) sets criteria for determining if a compound is orally active, with parameters like ALogP < 5, MW < 500, HBD < 5, and HBA < 10 [72]. The chemical spaces, based on physicochemical properties related to the Ro5 and Veber’s rule (i.e., nRot < 10 and TPSA < 140 Å^2^), were analyzed and depicted in Fig. 2. The findings reveal that the majority of compounds in the active group exhibit a MW ranging from 400 to 600 Da, while those in the inactive group are clustered within the 300–500 Da range (Fig. 2A). Similarly, the AlogP values for both active and inactive groups (Fig. 2B) depict compound density within the ranges of 4–6 and 3–5.5, respectively. Although these properties exhibit slight differences, they are statistically significant, as determined by the Mann–Whitney *U *Test with a *p*-value of < 0.05. The HBA and HBD parameters delineate the hydrogen bonding capacity of the compounds. Our results indicate that the majority of compounds in both classes meet the Ro5 criteria for HBA and HBD (Fig. 2C, D), although the differences between the groups are not statistically significant for the HBA property. Furthermore, the active and inactive compounds exhibit maximum ranges of 50–100 and 60–80 Å^2^ for TPSA, and 3–8 and 2–7 for nRotB, respectively (Fig. 2E, F). The differences between the classes for both properties are statistically significant, with *p*-values of < 0.05. Thus, it can be inferred that the statistical significance between these groups can provide insights into the relationship between the active compounds and their biological activity as inhibitors. This information can be valuable in guiding the design and optimization of new drug candidates.

To ensure that the independent test dataset is sufficiently distinct from the training dataset to avoid overestimation of model performance, we performed a scaffold analysis using the Bemis-Murcko framework [73] to demonstrate the number of unique scaffolds found in either the training dataset or independent test dataset. The scaffold analysis results showed that there are 50.23% of unique scaffolds found in the independent test dataset (detailed in Supplementary Table S1). In addition, to confirm the generalization ability of the proposed model, we computed the Tanimoto similarity coefficient between compound pairs in the training and independent test datasets based on ECFP4 fingerprints. As can be seen from Supplementary Figures S1-S2, the heatmap is predominantly blue, while the average Tanimoto similarity coefficient was 0.135 and 98.33% of compound pairs in the training and independent test datasets exhibited a Tanimoto similarity coefficient of less than 0.5, indicating low similarity between the training and independent test datasets.

### Effect of the under-sampling method on prediction performance

**Fig. 3:**
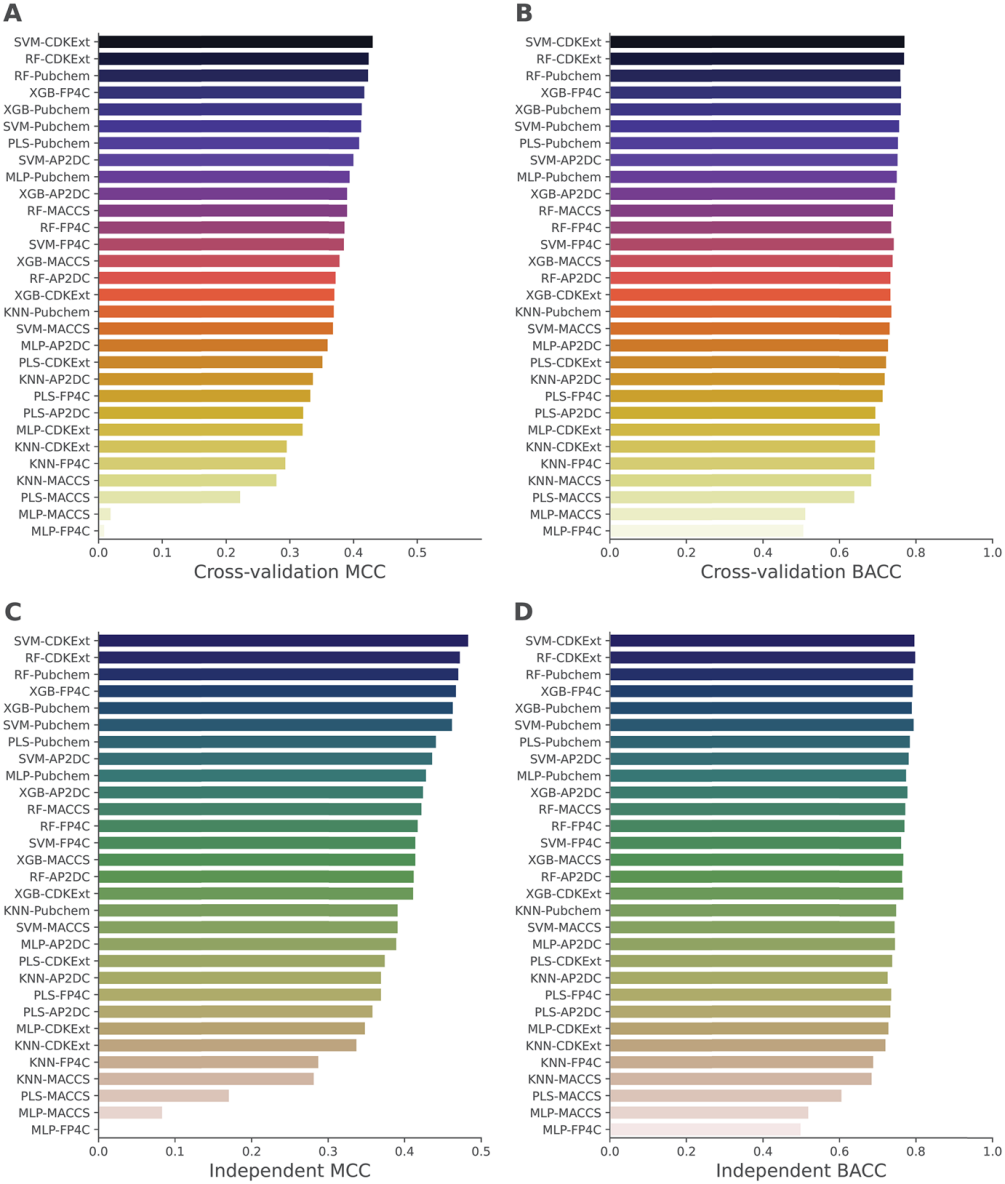
Average BACC and MCC scores of different ML classifiers as evaluated using the tenfold cross-validation (**A**, **B**) and independent tests (**C**, **D**) over the imbalanced datasets

This section investigated two different comparative scenarios. In the first scenario, we compared the performance of various ML classifiers trained with six ML algorithms coupled with five molecular descriptors on the imbalanced dataset. In the second scenario, these ML classifiers were trained and tested on the five balanced training subsets (i.e., BTS1–BTS5). To assess the contributions of molecular descriptors, ML algorithms, and under-sampling technique, six performance measures including BACC, AUC, SN, SP, MCC, and ACC achieved by the ML classifiers were evaluated and compared using ten-fold cross-validation and independent tests. As mentioned above, ML classifiers achieving the highest cross-validation MCC were regarded as the best-performing classifiers. The experimental results of these two comparative scenarios are provided in Fig. 3 and Tables 2 along with Supplementary Tables S3-S9.

**Table 2:** Cross-validation MCC scores of 30 base-classifiers developed based on individual five balanced training datasets

Descriptor	Method	BTS1	BTS2	BTS3	BTS4	BTS5	Average
AP2DC	KNN	0.509	0.342	0.446	0.441	0.470	0.442
	MLP	0.508	0.449	0.513	0.472	0.507	0.490
	PLS	0.498	0.425	0.404	0.398	0.501	0.445
	RF	0.482	0.487	0.513	0.455	0.513	0.490
	SVM	0.549	0.524	0.548	0.459	0.526	0.521
	XGB	0.540	0.494	0.533	0.497	0.457	0.504
CDKExt	KNN	0.469	0.421	0.431	0.400	0.487	0.442
	MLP	0.426	0.477	0.484	0.443	0.496	0.465
	PLS	0.365	0.474	0.464	0.478	0.472	0.451
	RF	0.450	0.519	0.494	0.522	0.521	0.501
	SVM	0.517	0.555	0.489	0.476	0.516	0.510
	XGB	0.483	0.479	0.444	0.472	0.516	0.479
FP4C	KNN	0.417	0.382	0.451	0.395	0.447	0.418
	MLP	0.007	0.031	0.050	-0.018	0.111	0.036
	PLS	0.436	0.434	0.400	0.341	0.452	0.413
	RF	0.478	0.509	0.519	0.480	0.545	0.506
	SVM	0.468	0.500	0.548	0.454	0.545	0.503
	XGB	0.520	0.539	0.503	0.495	0.560	0.524
MACCS	KNN	0.323	0.392	0.395	0.337	0.410	0.371
	MLP	0.017	0.040	-0.050	-0.023	-0.058	-0.015
	PLS	0.273	0.336	0.335	0.267	0.394	0.321
	RF	0.406	0.524	0.539	0.440	0.527	0.487
	SVM	0.339	0.510	0.493	0.405	0.443	0.438
	XGB	0.426	0.494	0.514	0.478	0.472	0.477
Pubchem	KNN	0.442	0.379	0.374	0.452	0.441	0.417
	MLP	0.468	0.469	0.404	0.546	0.511	0.480
	PLS	0.554	0.505	0.444	0.526	0.516	0.509
	RF	0.563	0.509	0.499	0.528	0.531	0.526
	SVM	0.491	0.460	0.451	0.563	0.526	0.498
	XGB	0.540	0.433	0.464	0.546	0.506	0.498

As can be seen from Supplementary Table S3, it can be noticed that there is no ML classifier trained with the imbalanced dataset achieving a cross-validation MCC value greater than 0.5. The top-five ML classifiers in this case were SVM-CDKExt, RF-CDKExt, RF-Pubchem, XGB-FP4C, and XGB-Pubchem with corresponding MCC scores of 0.431, 0.425, 0.424, 0.418, and 0.414, respectively (Fig. 3). In the meanwhile, on the independent test dataset, their performance remained unsatisfactory, with corresponding MCC scores of 0.418, 0.423, 0.429, 0.413, and 0.464, respectively (Supplementary Table S4). This indicated that the imbalanced dataset could be detrimental to model performance. Therefore, we were motivated to employ the under-sampling technique to improve model performance.

**Fig. 4:**
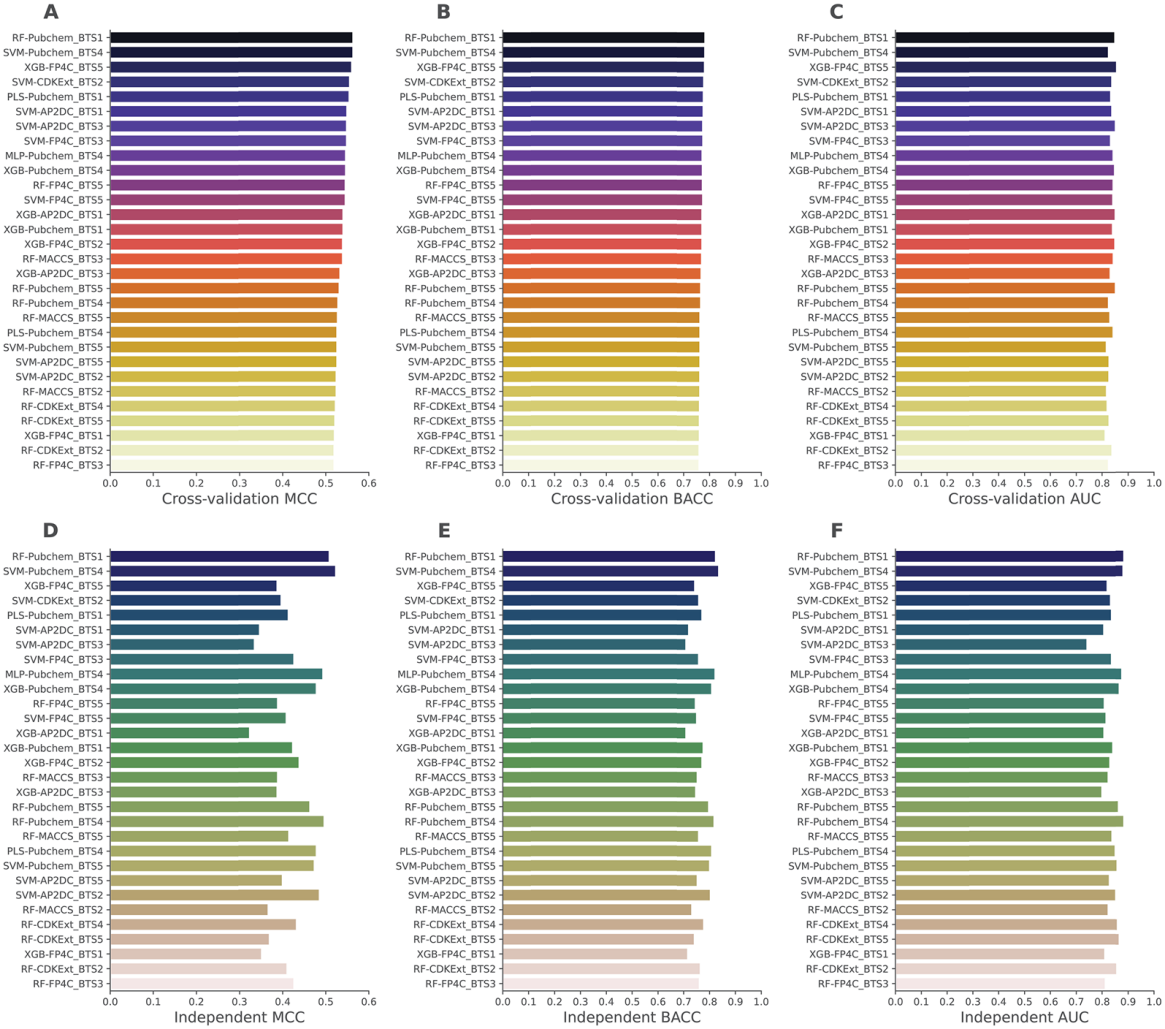
MCC, BACC, and AUC scores of top-30 ML classifiers trained with five balanced training subsets as evaluated using the tenfold cross-validation (**A**–**C**) and independent tests (**D**–**F**)

In case of the models trained with the balanced training subsets, it was observed that all of top-50 ML classifiers provided cross-validation MCC scores greater than 0.5 (Supplementary Table S5). These results demonstrate that the performance of the models trained based on balanced training subset are better than that of the imbalanced training dataset. We noticed that the top-five ML classifiers, achieving cross-validation MCC scores of 0.563, 0.563, 0.560, 0.555, and 0.554, were RF-Pubchem_BTS1, SVM-Pubchem_BTS4, XGB-FP4C_BTS5, SVM-CDKExt_BTS2, and PLS-Pubchem_BTS1, respectively (Fig. 4). Among these classifiers, two classifiers were able to attain MCC scores of 0.508 (RF-Pubchem_BTS1) and 0.523 (SVM-Pubchem_BTS4) as judged the independent test (Supplementary Table S6). To characterize the performance of the models, we calculated the average cross-validation and independent test MCC scores over the five balanced datasets with respect to each ML classifier. As shown in Table 2 and Supplementary Table S7, the top-five ML classifiers, achieving average cross-validation MCC scores of 0.526, 0.524, 0.521, 0.510, and 0.509 are RF-Pubchem, XGB-FP4C, SVM-AP2DC, SVM-CDKExt, and PLS-Pubchem, respectively. To confirm the effectiveness of the under-sampling technique, we compared the performance of the best-performing models trained with balanced (RF-Pubchem_BTS1) and imbalanced (SVM-CDKExt) training datasets. Additionally, we compared the performance of the top-five ML classifiers trained with their balanced datasets to that of the top-five ML classifiers trained with the imbalanced dataset. As shown in Supplementary Table S8, it is apparent that the top-five ML classifiers trained with their balanced datasets perform better than those trained with their imbalanced dataset in terms of BACC and MCC over the training subset. Furthermore, in the independent test, RF-Pubchem_BTS1 outperformed SVM-CDKExt, achieving a BACC of 0.823, MCC of 0.508, and AUC of 0.883 (Supplementary Table S9). This confirms again that the under-sampling technique is beneficial for enhancing model performance. Therefore, we utilized all the ML classifiers trained with the balanced dataset to construct our proposed models in the following studies.

### Construction of M3S-GRPred

Generally, it is straightforward to select the best ML classifiers among various ML classifiers trained with different ML algorithms and molecular descriptors. However, the predictive ability of single-feature-based models might not be robust enough [41, 47–49, 51, 55, 74]. To deal with the limitation arising from the single-feature-based models, we employed our powerful M3S method to develop the stacked ensemble learning model. Our stacked ensemble learning model was developed based on SVM method (referred to be mSVM) trained with the 150-D probabilistic feature vector. In addition, we applied the two-step feature selection method to identify *m* useful PFs. In this feature selection method, we initially ranked the PFs based on MDGI scores and generated 15 feature subsets containing *m* top-ranked informative PFs. After that, all the 15 feature subsets were used to develop different mSVM and their performance was assessed over both the cross-validation and independent tests**.** As shown in Supplementary Table S10, it is apparent that the feature subsets containing 140 and 150 top-ranking PFs achieved better performance than other feature subsets in term of MCC over the cross-validation test, which are referred as PFV and PFV_FS herein, respectively. The performance evaluation results of PFV and PFV_FS are recorded in Table 3. As can be seen, PFV and PFV_FS achieve similar cross-validation MCC scores of 0.713 and 0.708, respectively. On the independent test dataset, the MCC, SN, and BACC of PFV_FS were 0.658, 0.928, and 0.891, which were 1.48, 1.45, and 0.880% higher than PFV, respectively, demonstrating the effectiveness and robustness of PFV_FS. Therefore, we utilized PFV_FS to develop the final stacked ensemble learning model (M3S-GRPred).

**Table 3:** Performance of new feature representations over the ten-fold cross-validation and independent tests

Evaluation strategy	Feature	ACC	BACC	SN	SP	MCC	AUC
Cross-validation	PFV	0.900	0.903	0.908	0.898	0.713	0.965
	PFV_FS	0.898	0.900	0.903	0.897	0.708	0.964
Independent test	PFV	0.862	0.882	0.913	0.851	0.643	0.944
	PFV_FS	0.867	0.891	0.928	0.854	0.658	0.953

### M3S-GRPred outperforms several traditional machine learning-based classifiers

**Fig. 5:**
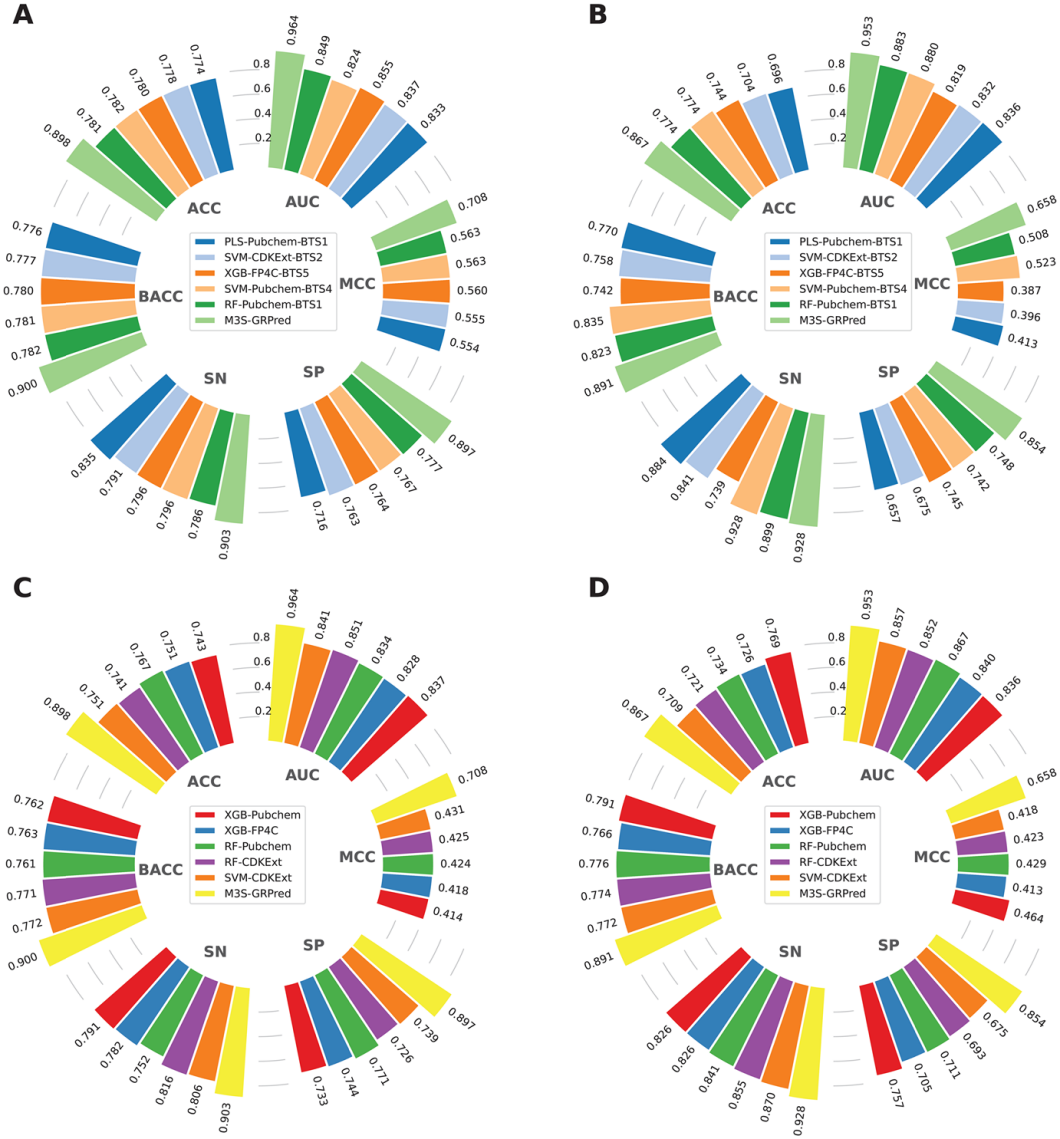
Performance comparison of M3S-GRPred and several conventional ML over the training (**A**, **C**) and independent (**B**, **D**) datasets. **A**, **B** ACC, BACC, SN, SP, MCC, and AUC of M3S-GRPred and top-five ML classifiers trained with the imbalanced datasets. **C**, **D** ACC, BACC, SN, SP, MCC, and AUC of M3S-GRPred and top-five ML classifiers trained with balanced training subsets

In this section, we compared the performance M3S-GRPred with its constituent base-classifiers trained with balanced and imbalanced training subsets to demonstrate the advantage of the M3S strategy in overcoming the data imbalance problem and attaining the performance improvement and robustness. In the first comparative experiment, we compared M3S-GRPred with the top-five base-classifiers trained with different balanced training subsets. As mentioned above, the top-five base-classifiers in this case were RF-Pubchem_BTS1, SVM-Pubchem_BTS4, XGB-FP4C_BTS5, SVM-CDKExt_BTS2, and PLS-Pubchem_BTS1. Figure 5A, B and Table 4 shows that M3S-GRPred exhibited better performance than the compared base-classifiers over the ten-fold cross-validation and independent tests. Specifically, on the independent test dataset, M3S-GRPredachieved remarkable improvements of 5.62–14.89, 13.46–27.12, and 6.95–13.33% in terms of BACC, MCC, and AUC, respectively. These results demonstrate that M3S-GRPredattained high accuracy and stability in the identification of GR antagonists. In addition, to determine whether our proposed framework can address the imbalanced data problem, we compared M3S-GRPredwith the top-five base-classifiers trained with the imbalanced training dataset. As illustrated in Fig. 5C, D and Table 5, significant improvements in prediction performances were observed across all six measures on both the independent test and training datasets. Specifically, our proposed model significantly improved BACC by 9.94–12.52%, MCC by 19.35–24.51%, and AUC by 8.57–11.26%. Altogether, these results indicate that our proposed framework used to develop M3S-GRPrednot only addresses the data imbalance problem, but also effectively leverages heterogeneous models to achieve more stable and accurate identification of GR antagonists.

**Table 4:** Performance comparison of M3S-GRPred and top-five ML base-classifiers developed using different balanced training subsets

Evaluation strategy	Method	ACC	BACC	SN	SP	MCC	AUC
Cross-validation	PLS-Pubchem-BTS1	0.774	0.776	0.835	0.716	0.554	0.833
	SVM-CDKExt-BTS2	0.778	0.777	0.791	0.763	0.555	0.837
	XGB-FP4C-BTS5	0.780	0.780	0.796	0.764	0.560	0.855
	SVM-Pubchem-BTS4	0.782	0.781	0.796	0.767	0.563	0.824
	RF-Pubchem-BTS1	0.781	0.782	0.786	0.777	0.563	0.849
	M3S-GRPred	0.898	0.900	0.903	0.897	0.708	0.964
Independent test	PLS-Pubchem-BTS1	0.696	0.770	0.884	0.657	0.413	0.836
	SVM-CDKExt-BTS2	0.704	0.758	0.841	0.675	0.396	0.832
	XGB-FP4C-BTS5	0.744	0.742	0.739	0.745	0.387	0.819
	SVM-Pubchem-BTS4	0.774	0.835	0.928	0.742	0.523	0.880
	RF-Pubchem-BTS1	0.774	0.823	0.899	0.748	0.508	0.883
	M3S-GRPred	0.867	0.891	0.928	0.854	0.658	0.953

**Table 5:** Performance comparison of M3S-GRPred and top-five base-classifiers developed using the imbalanced training subset

Evaluation strategy	Method	ACC	BACC	SN	SP	MCC	AUC
Cross-validation	XGB-Pubchem	0.743	0.762	0.791	0.733	0.414	0.837
	XGB-FP4C	0.751	0.763	0.782	0.744	0.418	0.828
	RF-Pubchem	0.767	0.761	0.752	0.771	0.424	0.834
	RF-CDKExt	0.741	0.771	0.816	0.726	0.425	0.851
	SVM-CDKExt	0.751	0.772	0.806	0.739	0.431	0.841
	M3S-GRPred	0.898	0.900	0.903	0.897	0.708	0.964
Independent test	XGB-Pubchem	0.769	0.791	0.826	0.757	0.464	0.836
	XGB-FP4C	0.726	0.766	0.826	0.705	0.413	0.840
	RF-Pubchem	0.734	0.776	0.841	0.711	0.429	0.867
	RF-CDKExt	0.721	0.774	0.855	0.693	0.423	0.852
	SVM-CDKExt	0.709	0.772	0.870	0.675	0.418	0.857
	M3S-GRPred	0.867	0.891	0.928	0.854	0.658	0.953

### Model interpretation and feature importance analysis

To gain deeper insight into specific substructural elements potentially responsible for antagonistic effects against GR, we employed the RF classifier to determine and rank the feature importance based on the MDGI [43–45, 50, 75]. Since the top-three base-classifiers were developed using BTS1, BTS4, and BTS5, we performed feature importance analysis using RF classifiers couple with an interpretable feature descriptor (i.e., Pubchem) on these three balanced training subsets. The important features identified in BTS1, BTS4, and BTS5 are summarized in Table 6. Features with the highest MDGI scores are considered most important for GR antagonist identification. Consequently, we selected the top-20 important features found in all three balanced training subsets for detailed feature importance analysis, as summarized in Table 6. Taking Pubchem568 as an example, its MDGI scores (ranks) based on BTS1, BTS4, and BTS5 were 3.23(1), 1.22(8), and 1.46(9), respectively.

**Table 6:** List of important Pubchem-based features along with their MDGI scores (ranks) found in all three balanced training datasets (i.e., BTS1, BTS5, and BTS4)

Feature	BTS1	BTS4	BTS5	SMARTS pattern	Description
Pubchem568	3.23(1)	1.22(8)	1.46(9)	N#C–C–C	Propionitrile
Pubchem799	2.59(3)	4.65(1)	3.57(1)	CC1CC(S)CCC1	3-methylcyclohexane-1-thiol
Pubchem663	2.57(4)	0.99(19)	1.15(18)	O–C–C–O-[#1]	Ethylene glycol
Pubchem4	1.67(7)	1.14(11)	1.52(8)	> = 1 Li	greater than or equal to one Lithium atom
Pubchem778	1.67(8)	1.15(10)	1.62(6)	CC1CCC(S)CC1	4-Methylcyclohexane-1-thiol
Pubchem736	1.65(9)	2.35(2)	1.93(3)	Cc1cc(S)ccc1	3-Methylbenzenethiol
Pubchem682	1.41(10)	2.12(3)	1.57(7)	O–C–C–C–C–N	4-Amino-1-butanol
Pubchem340	1.34(12)	1.08(15)	1.23(14)	C(~ C)(~ C)(~ N)	Isopropylamine
Pubchem193	1.33(13)	1.09(14)	2.07(2)	> = 3 saturated or aromatic carbon-only ring size 6	greater than or equal to 3 saturated or aromatic carbon-only ring of size six
Pubchem336	1.13(18)	1.16(9)	1.16(17)	C(~ C)(~ C)(~ C)(~ N)	2-methylpropan-2-amine

From our analysis, it was highlighted that the top feature in BTS4 and BTS5 were the same, i.e., Pubchem799 (2.59(3), 4.65(1), 3.57(1)) which pertains to 3-methylcyclohexane-1-thiol. This compound is an alkylthiol, meaning that an alkyl group (i.e., methylcyclohexane) is attached to a sulfhydryl group. In addition, Pubchem736 (1.65(9), 2.35(2), 1.93(3)) and Pubchem778 (1.67(8), 1.15(10), 1.62(6)), corresponding to 3-methylbenzenethiol and 4-methylcyclohexane-1-thiol, respectively, are among the top 10 features containing alkylthiol substructures. The thiol (− SH) functional group is found in numerous drug compounds, imparting a unique combination of useful properties. Thiol-containing drugs act as antioxidants by neutralizing radicals and other harmful electrophiles, replenishing cellular thiol pools, and forming stable complexes with heavy metals like arsenic, copper, and lead [76]. In addition, thiol-based drugs are classified as mucolytics due to their ability to lower the thickness and flexibility of bronchial secretions by breaking down disulfide bonds in proteins [77]. A recent study by Khanna et al. [78], explored the antiviral and anti-inflammatory effects of thiol-based drugs in Covid-19. The authors observed that in vivo treatment with thiol drugs (i.e., cysteamine) exerted anti-inflammatory effects and reduced SARS-CoV-2-induced lung inflammation and injury. Moreover, in vitro assays showed that multiple thiol drugs were capable of inhibiting the binding of SARS-CoV2 spike protein to its receptor thereby, inhibiting viral infection [78]. Taken together, the anti-inflammatory and antioxidant properties of thiol-based drugs could be beneficial for treating Cushing’s syndrome, as GR is associated with the inflammation pathway.

Pubchem568 (3.23(1), 1.22(8), 1.46(9)) corresponds to propionitrile, an aliphatic nitrile, polar aprotic solvent, and a natural product found in the Apis species [79]. Additionally, propionitrile serves as a precursor for diarylpropionitrile (DPN), which exhibits strong ERβ agonist properties [80]. Furthermore, DPN has demonstrated antidepressant- and anxiolytic-like effects in animals by activating the endogenous oxytocin system, the body’s natural mechanism for managing stress and promoting well-being [81]. A study by Thangnipon et al., assessed the neuroprotective effects of DPN against oxidative stress in human neuroblastoma cells and concluded that DPN could be beneficial for protecting against neurodegenerative diseases [82]. Several studies have also investigated the role of DPN in breast cancer inhibition [83, 84]. Given that GR belongs to the same superfamily as ERβ (i.e., steroid nuclear receptors), exploring the effects of DPN on GR is worthwhile.

Pubchem340 (1.34(12), 1.08(15), 1.23(14)) and Pubchem336 (1.13(18), 1.16(9), 1.16(17)) corresponding to isopropylamine and 2-methylpropan-2-amine (i.e., tert-Butylamine), respectively are primary aliphatic amines found in the top-ten common features. These compounds serve as prodrug moieties in medicinal chemistry, particularly valued for their efficient drug release capabilities with both small and long-chain aliphatic amines [85]. Moreover, the substitution of aliphatic amines in GR modulators have been patented (US20060154973A1 and WO2005090336A1), exploring a new class of non-steroidal compound for treating GR-associated diseases. Isopropylamine (Pubchem340), when combined with 2-chloro-1-(3,4-dihydroxyphenyl)ethan-1-one, serves as a precursor for sympathomimetic β-adrenoreceptor drugs such as isoprenaline and metaproterenol, used in COPD and asthma treatment [86]. Similarly, the synthesis of salbutamol, a short-acting β2 adrenergic agonist used to treat asthma and COPD, begins with the acylation of salicylaldehyde to form a α-bromo acetophenone derivative. This intermediate then reacts with tert-butylamine (Pubchem336) in isopropanol [86]. The tert-butyl group from tert-butylamine is highly lipophilic and introduces steric hindrance in the inhibitor molecule, which may advantageously block specific binding sites and prevent interaction of the GR with endogenous glucocorticoids or other ligands. Thus, the aliphatic amine functionality of these features could potentially form hydrogen bonds or participate in other non-covalent interactions with the GR.

### Case study: prospective GR inhibitors from FDA-approved drugs

#### Identification of potential GR inhibitors

In this section, we firstly employed M3S-GRPred for virtual screening, where the model was used to estimate the probability score for each FDA-approved compound from DrugBank. Secondly, the top 30 compounds with the highest probability scores were selected and subjected to molecular docking for predicting binding affinity to GR. Thirdly, among these, four potential compounds were selected for MD simulations to examine ligand stability in dynamic conditions using free energy calculations. (details in the Materials and Methods section). Supplementary Table S11 lists the top compounds with their probabilities, corresponding docking scores, and inhibitor target sites. Notably, the top-four compounds with the highest docking scores are all inhibitors of PR or related nuclear receptors, which have demonstrated cross interactions with GR. Therefore, in efforts for drug repurposing, these inhibitors were not considered for further MD simulations. The selected four compounds were elucidated to be azelastine (AZE), metergoline (MET), perampanel (PER), and pirenzepine (PIR), with docking scores of − 9.3, − 8.8, − 8.7, and − 8.3 kcal/mol, respectively. These compounds, along with the reference co-crystal compound (i.e., Mifepristone (MF), − 11.4 kcal/mol), which is the only FDA-approved GR antagonist, underwent further evaluation through MD simulations.

#### Structural dynamics and binding affinity of screened drugs against GR

**Fig. 6:**
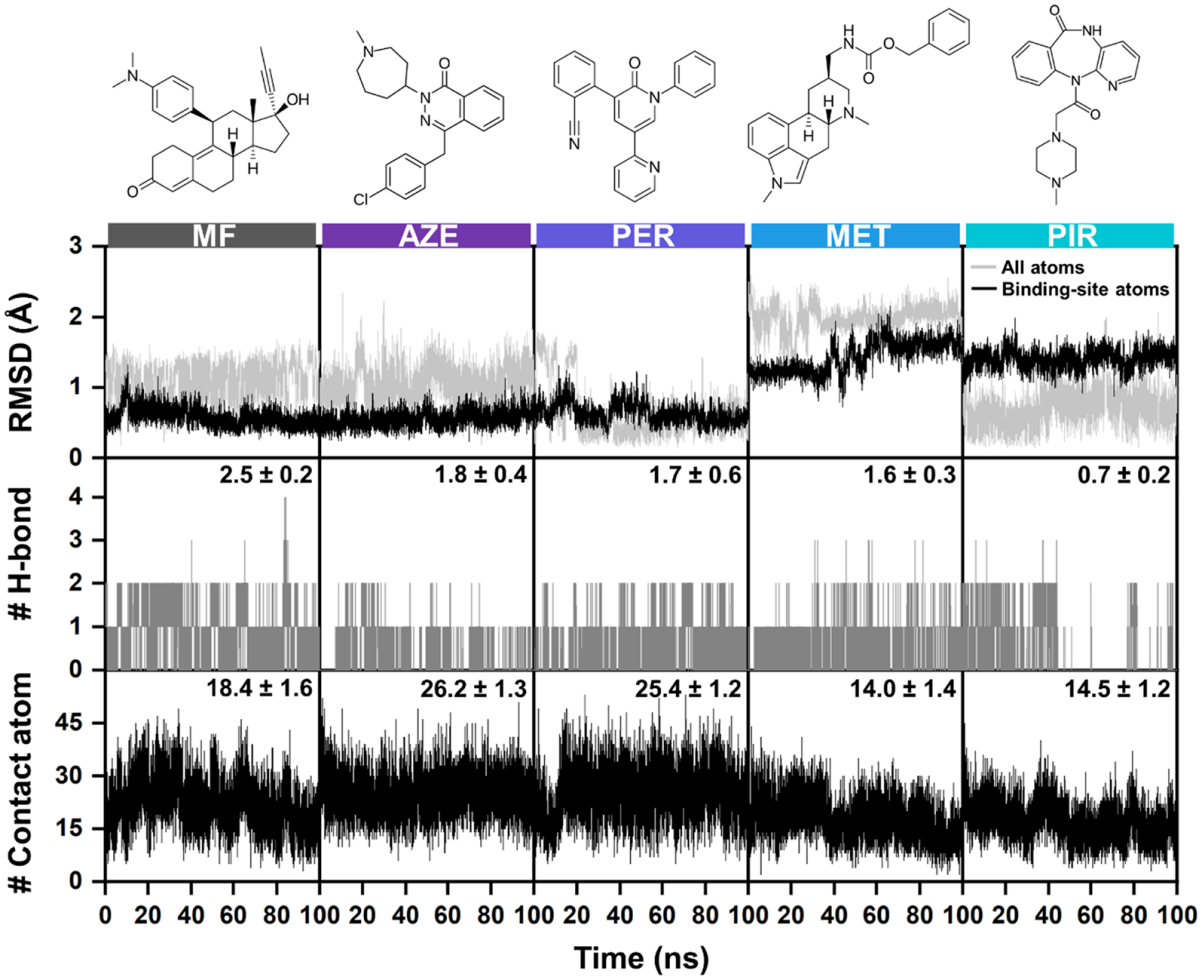
All-atom RMSD, # H-bonds, and # atom contacts of mifepristone (MF) and the selected drugs such as, azelastine (AZE), perampanel (PER), metergoline (MET), and pirenzepine (PIR) in complex with GR target plotted over a 100-ns duration of run1-MD simulations. The corresponding data for run2 and run3 simulations can be found in Supplementary Figures S1 and S2, respectively. The values shown in the figure are the averages calculated from the last 20 ns of three independent MD simulations

**Fig. 7:**
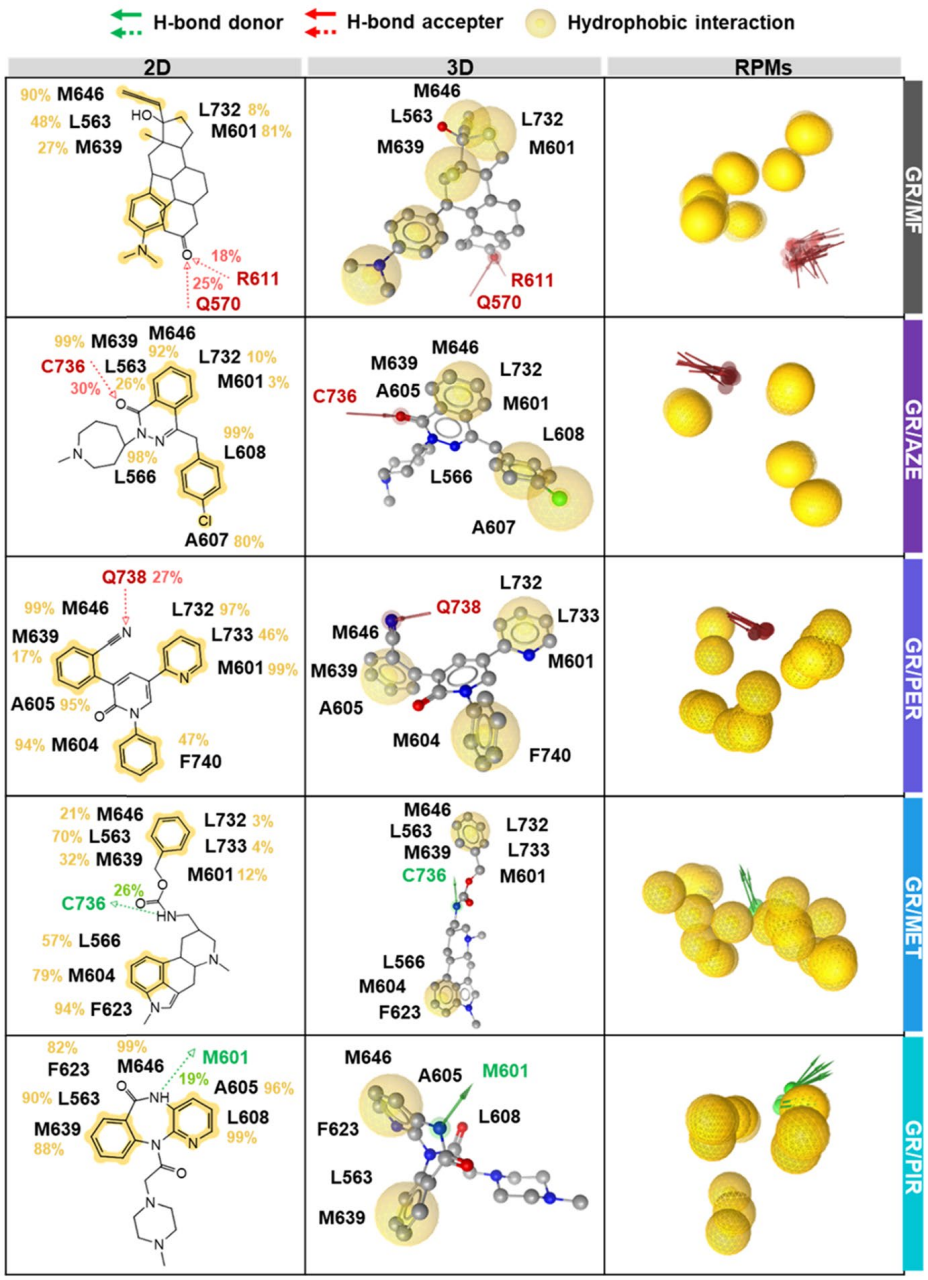
The crucial interactions of MF, AZE, PER, MET and PIR in complex with GR are depicted in 2D and 3D pharmacophore models, along with the representative pharmacophore models (RPMs) analyzed from the last 20 ns of MD simulations. The green arrow, red arrow, and yellow circle represent the pharmacophore features of hydrogen bond donor (HBD), hydrogen bond acceptor (HBA), and hydrophobic interaction, respectively

The stability of ligand-binding was assessed using plots of root mean square deviation (RMSD), the number of intermolecular hydrogen bonds (# H-bonds), and the number of atom contacts (# atom contacts) against simulation time. Figure 6 and Supplementary Figures S3 and S4 illustrate that all atoms in the five drug/GR complex systems exhibited high fluctuation during MD simulations; however, the binding sites of all five systems showed less variability. Based on this observation and the plots of # H-bonds and # atom contacts, all simulated systems reached equilibrium at 40 ns. Therefore, in this study, snapshots from the final 20 ns were selected for further analysis in terms of binding free energy and drug/protein interactions, as depicted in Figs. 6 and 7, respectively.

As observed in Fig. 6, each focused drug formed 1.6–1.8 H-bonds with the GR protein target, except for PIR (0.7). Meanwhile, the # atom contacts reached ~ 25 in the complexes bound with AZE and PER, which was higher compared to the reference drug MF (~ 18). Therefore, van der Waals (vdW) interactions could serve as the predominant contributor for drug binding. Interestingly, the # atom contacts in the rest of the complexes (~ 14) were somewhat lower than those in the MF system. Additionally, the ΔGbind values obtained from MM/GBSA for all systems followed a similar trend to those from MM/PBSA, as shown in Supplementary Figure S5. AZE and PER exhibited binding energies with GR in the same magnitude as MF. Furthermore, the # H-bonds in MF/GR complex (2.5 ± 0.2, Fig. 6) involving Q570 and R611 (25% and 18%, Fig. 7) corresponded with previous findings [59], slightly exceeding those in AZE (1.8 ± 0.4) with C736 (30%), PER (1.7 ± 0.6) with Q738 (27%), MET (1.6 ± 0.3) with C736 (26%), and PIR (0.7 ± 0.2) with M601 (19%). However, the binding strengths of the potential drug candidates AZE and PER, were compensated by their higher # atom contacts (26.2 ± 1.3) and (25.4 ± 1.2), respectively, compared to MF (18.4 ± 1.6).

According to Fig. 7, both effective drugs showed potential hydrophobic interactions with residues L566 (98%), M604 (94%), A605 (95%), A607 (80%), L608 (99%), L733 (46%), and F740 (47%), whereas MF interacted only with M464 (90%), L563 (48%), M601 (81%), M639 (27%), and L732 (8%) through hydrophobic interactions. Additionally, the MM energies confirmed that vdW interactions, assessed by MM/GBSA and MM/PBSA, are the primary force driving molecular complexation, with values of − 61.1 ± 0.4 and − 61.3 ± 0.4 kcal/mol, respectively, for AZE, as well as − 61.1 ± 0.4 and − 60.7 ± 0.4 kcal/mol, respectively, for PER (Supplementary Table S12). Taken together, the results of ligand–protein interactions, coupled with the obtained binding affinities, suggest that AZE and PER could potentially serve as GR antagonist similar to MF.

## Conclusions

We have developed M3S-GRPred, a novel ensemble learning framework utilizing the stacking strategy to rapidly and accurately discover novel GR antagonists using only SMILES information. M3S-GRPred first constructs balanced training subsets via under-sampling, then employs these subsets to train heterogeneous base classifiers with various SMILES-based feature descriptors and ML algorithms. The final model integrates probabilistic outputs from these base classifiers. To reveal the effectiveness of the proposed M3S-GRPred model, we compared its performance with several conventional ML classifiers over the ten-fold cross-validation and independent tests. Our comparative results shows that M3S-GRPred significantly outperforms conventional ML classifiers, achieving a balanced accuracy of 0.891, MCC of 0.658, and AUC of 0.953, with improvements of 9.94–12.52%, 19.35–24.51%, and 8.57–11.26%, respectively, on an independent test dataset. It also successfully identified potential GR antagonists among FDA-approved drugs, confirmed through molecular docking and MD simulation studies for drug repurposing in Cushing’s syndrome. We anticipate that M3S-GRPred will be an efficient screening tool for discovering novel GR antagonists cost-effectively from vast libraries of unknown compounds.

## Supplementary Information

Supplementary material 1

## Data Availability

The molecular data used in this research were acquired from the ChEMBL database version 33 with target ID: CHEMBL2034 (https://www.ebi.ac.uk/chembl/search_results/CHEMBL2034). The case study dataset was acquired from the DrugBank database (version 5.1.10; released on January 4, 2023) of FDA-approved drugs (https://go.drugbank.com/). The implementation of this research and the R source codes are available at https://github.com/Shoombuatong/M3S-GRPred.
