# Supplementary material for: M3S-GRPred: a novel ensemble learning approach for the interpretable prediction of glucocorticoid receptor antagonists using a multi-step stacking strategy
Source: BMC Bioinformatics. 2025 Apr 30;26:117. doi: 10.1186/s12859-025-06132-1 (PMC12044944; doi:10.1186/s12859-025-06132-1)
Supplement: Supplementary file 1 — Supplementary material 1 [file 12859_2025_6132_MOESM1_ESM.docx]

## **Supplementary information**

**Total Scaffold Diversity:** measures how diverse the scaffolds are within each dataset, where a higher value indicates a more diverse dataset. It can be calculated by:

Scaffold Diversity (%) = (N/M)×100

where:

𝑁 = Number of unique scaffolds

𝑀 = Total number of compounds in the dataset

**Novel Scaffold Percentage**: measures the percentage of scaffolds in the independent test dataset that are absent in the training dataset. It can be calculated by:

Novel Scaffold (%) = (N_novel_/N_test_)×100

where:

𝑁_novel_ = Number of scaffolds present only in the independent test dataset

𝑁_test_ = Total number of unique scaffolds in the independent test dataset

*Bemis-Murcko scaffold was calculated using RDKit package in the Python programming environment.

## **Supplementary Figure**

## **Figure S1.** Heat map of Tanimoto coefficient between pairs of compounds in the training and independent test datasets based on ECFP4 fingerprints.


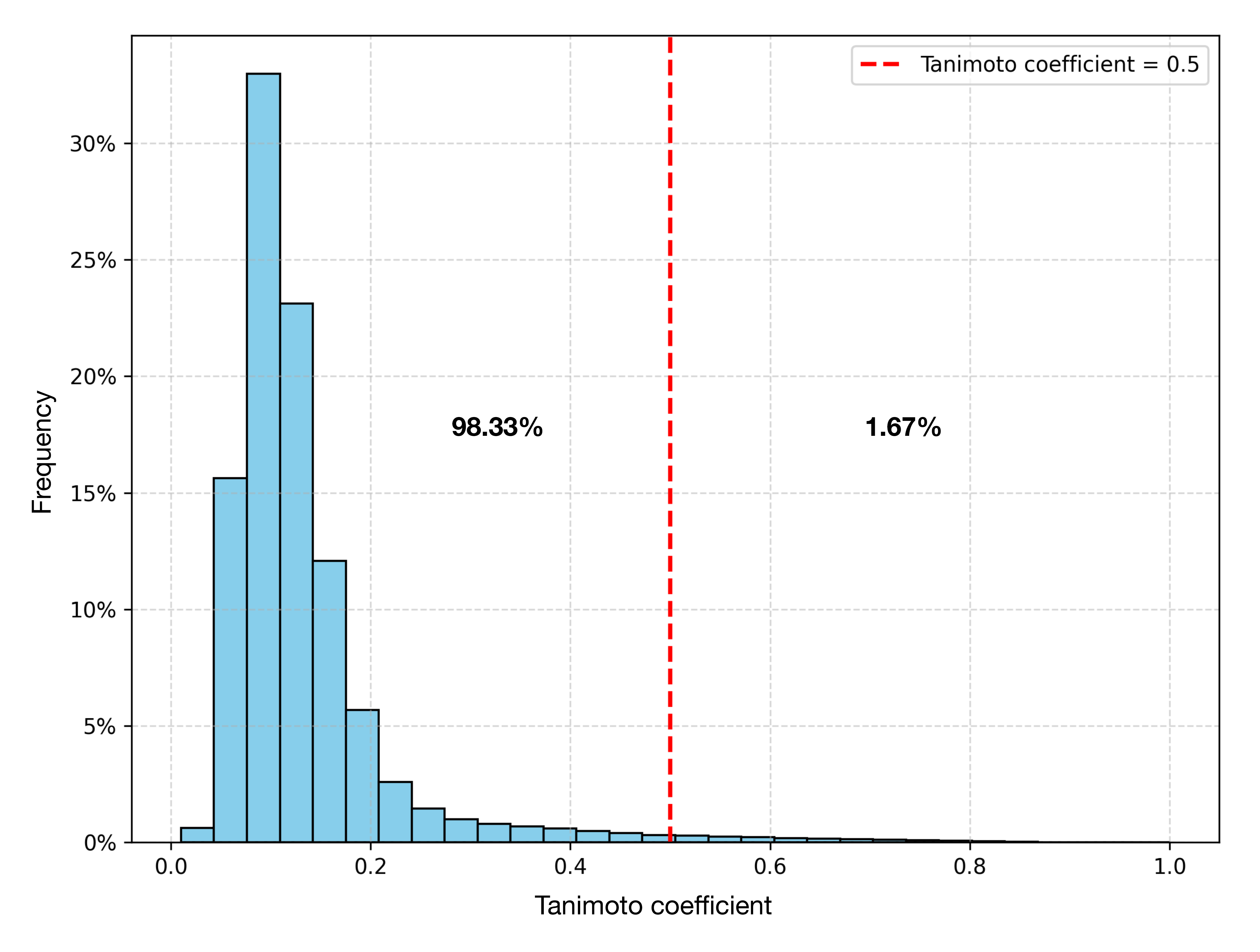


## **Figure S2.** Frequency histogram of Tanimoto similarity coefficient with ECFP4 fingerprints of compounds between training and independent test datasets.

**
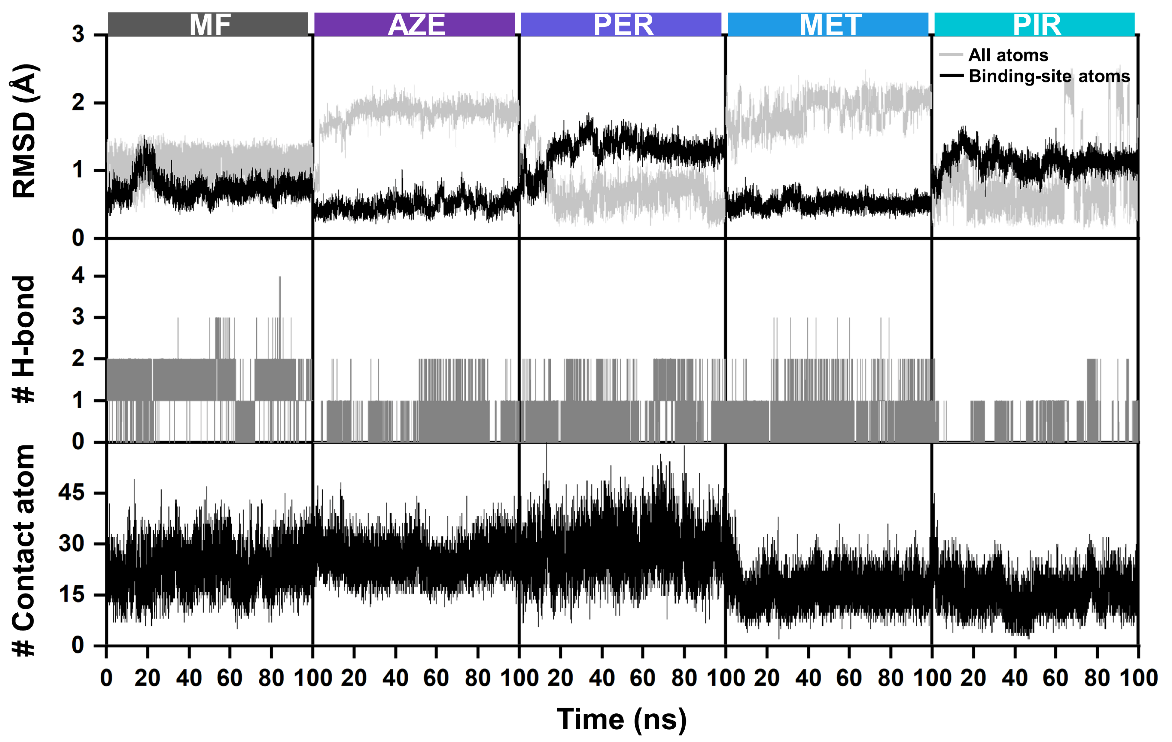
**

## **Figure S3.** All-atom RMSD, # H-bonds, and # atom contacts of mifepristone (MF) and the selected drugs such as, azelastine (AZE), perampanel (PER), metergoline (MET), and pirenzepine (PIR) in complex with GR target plotted over a 100-ns duration of run2-MD simulations.


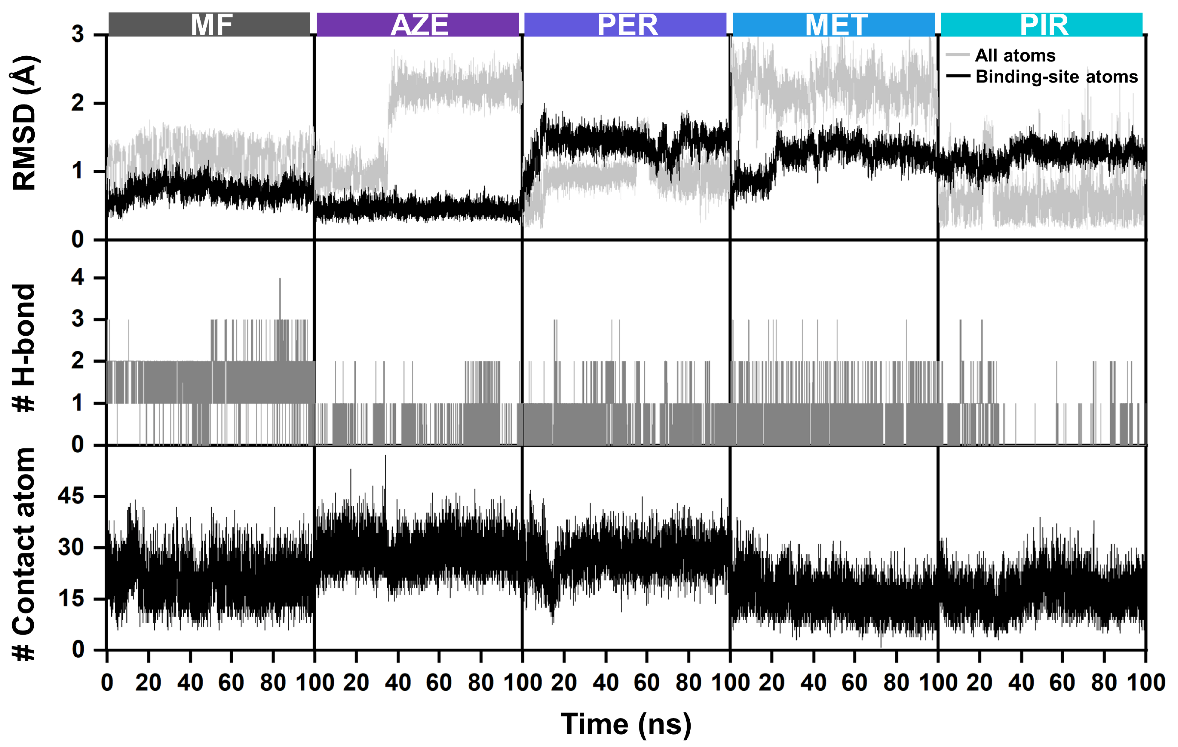


## **Figure S4.** All-atom RMSD, # H-bonds, and # atom contacts of mifepristone (MF) and the selected drugs such as, azelastine (AZE), perampanel (PER), metergoline (MET), and pirenzepine (PIR) in complex with GR target plotted over a 100-ns duration of run3-MD simulations.


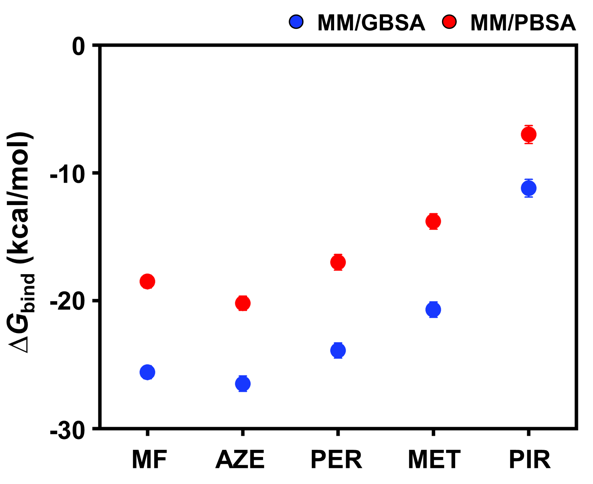


## **Figure S5.** ∆*G*_bind_ of all studied drugs/GR systems calculated with MM/GBSA and MM/PBSA strategies.

## **Supplementary Table**

## **Table S1** Scaffold analysis of compounds in the training and independent test datasets based on Bemis-Murcko framework.

| Dataset | Number of compounds **(M)** | Number of unique Murcko scaffolds  **(N)** | Scaffold diversity  **(N/M)*100** | Number of novel scaffolds **(N_novel_)** |
| --- | --- | --- | --- | --- |
| Training dataset | 1190 | 488 | 41% | 382 |
| Independent test set | 399 | 213 | 53% | 107 |

## **Table S2** Hyperparameter search details for six different ML classifiers.

| **Method** | **Parameters** | **Range of parameters** |
| --- | --- | --- |
| ET | n_estimators | [20, 50, 100, 200, 500] |
| KNN | number of neighbours | Default |
| LR | Cost | [0.001, 0.01, 0.1, 1, 10, 100] |
| PLS | #Components | Default |
| RF | n_estimators | [20, 50, 100, 200, 500] |
| SVM | Cost | [1, 2, 4, 8, 16, 32] |

Columns 2 and 3 represents the parameter name used in the Scikit-learn library and the range of parameter used to develop the model, respectively.

## **Table S3** Cross-validation results of different base-classifiers developed using six ML algorithms and five feature descriptors on the imbalanced training dataset.

| **Descriptor** | **Method** | **ACC** | **BACC** | **SN** | **SP** | **MCC** | **AUC** |
| --- | --- | --- | --- | --- | --- | --- | --- |
| AP2DC | KNN | 0.677 | 0.720 | 0.786 | 0.654 | 0.337 | 0.827 |
|  | MLP | 0.720 | 0.729 | 0.743 | 0.716 | 0.360 | 0.801 |
|  | PLS | 0.739 | 0.696 | 0.631 | 0.761 | 0.322 | 0.765 |
|  | RF | 0.733 | 0.735 | 0.738 | 0.732 | 0.373 | 0.829 |
|  | SVM | 0.739 | 0.754 | 0.777 | 0.731 | 0.401 | 0.827 |
|  | XGB | 0.737 | 0.747 | 0.762 | 0.732 | 0.391 | 0.836 |
| CDKExt | KNN | 0.644 | 0.695 | 0.772 | 0.617 | 0.296 | 0.803 |
|  | MLP | 0.690 | 0.707 | 0.733 | 0.681 | 0.321 | 0.784 |
|  | PLS | 0.715 | 0.724 | 0.738 | 0.711 | 0.352 | 0.803 |
|  | RF | 0.741 | 0.771 | 0.816 | 0.726 | 0.425 | 0.851 |
|  | SVM | 0.751 | 0.772 | 0.806 | 0.739 | 0.431 | 0.841 |
|  | XGB | 0.730 | 0.735 | 0.743 | 0.727 | 0.371 | 0.821 |
| FP4C | KNN | 0.657 | 0.693 | 0.748 | 0.638 | 0.294 | 0.820 |
|  | MLP | 0.428 | 0.507 | 0.626 | 0.387 | 0.010 | 0.851 |
|  | PLS | 0.696 | 0.715 | 0.743 | 0.686 | 0.333 | 0.769 |
|  | RF | 0.759 | 0.737 | 0.704 | 0.771 | 0.387 | 0.821 |
|  | SVM | 0.736 | 0.744 | 0.757 | 0.731 | 0.386 | 0.802 |
|  | XGB | 0.751 | 0.763 | 0.782 | 0.744 | 0.418 | 0.828 |
| MACCS | KNN | 0.634 | 0.685 | 0.762 | 0.607 | 0.280 | 0.817 |
|  | MLP | 0.622 | 0.512 | 0.345 | 0.680 | 0.020 | 0.548 |
|  | PLS | 0.676 | 0.641 | 0.587 | 0.694 | 0.223 | 0.707 |
|  | RF | 0.754 | 0.742 | 0.723 | 0.760 | 0.391 | 0.827 |
|  | SVM | 0.730 | 0.733 | 0.738 | 0.728 | 0.369 | 0.779 |
|  | XGB | 0.725 | 0.741 | 0.767 | 0.716 | 0.379 | 0.821 |
| Pubchem | KNN | 0.710 | 0.738 | 0.782 | 0.695 | 0.370 | 0.850 |
|  | MLP | 0.727 | 0.752 | 0.791 | 0.714 | 0.395 | 0.826 |
|  | PLS | 0.757 | 0.755 | 0.752 | 0.757 | 0.410 | 0.830 |
|  | RF | 0.767 | 0.761 | 0.752 | 0.771 | 0.424 | 0.834 |
|  | SVM | 0.755 | 0.758 | 0.762 | 0.753 | 0.413 | 0.814 |
|  | XGB | 0.743 | 0.762 | 0.791 | 0.733 | 0.414 | 0.837 |

## **Table S4** Independent test results of different base-classifiers developed using six ML algorithms and five feature descriptors on the imbalanced training dataset.

| **Descriptor** | **Method** | **ACC** | **BACC** | **SN** | **SP** | **MCC** | **AUC** |
| --- | --- | --- | --- | --- | --- | --- | --- |
| AP2DC | KNN | 0.623 | 0.686 | 0.783 | 0.590 | 0.282 | 0.824 |
|  | MLP | 0.729 | 0.750 | 0.783 | 0.717 | 0.392 | 0.834 |
|  | PLS | 0.741 | 0.763 | 0.797 | 0.729 | 0.415 | 0.797 |
|  | RF | 0.749 | 0.796 | 0.870 | 0.723 | 0.463 | 0.862 |
|  | SVM | 0.771 | 0.793 | 0.826 | 0.760 | 0.468 | 0.826 |
|  | XGB | 0.789 | 0.798 | 0.812 | 0.784 | 0.484 | 0.856 |
| CDKExt | KNN | 0.648 | 0.730 | 0.855 | 0.605 | 0.349 | 0.848 |
|  | MLP | 0.704 | 0.769 | 0.870 | 0.669 | 0.412 | 0.827 |
|  | PLS | 0.676 | 0.735 | 0.826 | 0.644 | 0.359 | 0.819 |
|  | RF | 0.721 | 0.774 | 0.855 | 0.693 | 0.423 | 0.852 |
|  | SVM | 0.709 | 0.772 | 0.870 | 0.675 | 0.418 | 0.857 |
|  | XGB | 0.731 | 0.786 | 0.870 | 0.702 | 0.442 | 0.860 |
| FP4C | KNN | 0.663 | 0.722 | 0.812 | 0.632 | 0.338 | 0.862 |
|  | MLP | 0.173 | 0.500 | 1.000 | 0.000 | 0.000 | 0.858 |
|  | PLS | 0.749 | 0.728 | 0.696 | 0.760 | 0.370 | 0.803 |
|  | RF | 0.734 | 0.747 | 0.768 | 0.726 | 0.390 | 0.815 |
|  | SVM | 0.774 | 0.795 | 0.826 | 0.763 | 0.471 | 0.851 |
|  | XGB | 0.726 | 0.766 | 0.826 | 0.705 | 0.413 | 0.840 |
| MACCS | KNN | 0.621 | 0.690 | 0.797 | 0.584 | 0.288 | 0.815 |
|  | MLP | 0.206 | 0.520 | 1.000 | 0.040 | 0.084 | 0.747 |
|  | PLS | 0.663 | 0.607 | 0.522 | 0.693 | 0.171 | 0.713 |
|  | RF | 0.716 | 0.737 | 0.768 | 0.705 | 0.370 | 0.830 |
|  | SVM | 0.721 | 0.740 | 0.768 | 0.711 | 0.375 | 0.807 |
|  | XGB | 0.741 | 0.746 | 0.754 | 0.739 | 0.392 | 0.824 |
| Pubchem | KNN | 0.726 | 0.783 | 0.870 | 0.696 | 0.437 | 0.882 |
|  | MLP | 0.656 | 0.780 | 0.971 | 0.590 | 0.425 | 0.858 |
|  | PLS | 0.714 | 0.769 | 0.855 | 0.684 | 0.415 | 0.834 |
|  | RF | 0.734 | 0.776 | 0.841 | 0.711 | 0.429 | 0.867 |
|  | SVM | 0.764 | 0.800 | 0.855 | 0.745 | 0.473 | 0.861 |
|  | XGB | 0.769 | 0.791 | 0.826 | 0.757 | 0.464 | 0.836 |

## **Table S5** Cross-validation results of 30 base-classifiers developed based on individual five training balanced datasets (i.e., BTS1–BTS5).

| **Dataset** | **Descriptor** | **Method** | **ACC** | **BACC** | **SN** | **SP** | **MCC** | **AUC** |
| --- | --- | --- | --- | --- | --- | --- | --- | --- |
| BTS1 | AP2DC | KNN | 0.751 | 0.752 | 0.825 | 0.679 | 0.509 | 0.862 |
|  | AP2DC | MLP | 0.753 | 0.754 | 0.782 | 0.726 | 0.508 | 0.836 |
|  | AP2DC | PLS | 0.748 | 0.749 | 0.772 | 0.726 | 0.498 | 0.825 |
|  | AP2DC | RF | 0.741 | 0.741 | 0.748 | 0.735 | 0.482 | 0.844 |
|  | AP2DC | SVM | 0.774 | 0.775 | 0.786 | 0.763 | 0.549 | 0.837 |
|  | AP2DC | XGB | 0.770 | 0.770 | 0.791 | 0.749 | 0.540 | 0.850 |
|  | CDKExt | KNN | 0.732 | 0.733 | 0.796 | 0.670 | 0.469 | 0.837 |
|  | CDKExt | MLP | 0.713 | 0.713 | 0.738 | 0.688 | 0.426 | 0.787 |
|  | CDKExt | PLS | 0.682 | 0.682 | 0.714 | 0.651 | 0.365 | 0.786 |
|  | CDKExt | RF | 0.724 | 0.725 | 0.752 | 0.698 | 0.450 | 0.835 |
|  | CDKExt | SVM | 0.758 | 0.758 | 0.782 | 0.735 | 0.517 | 0.816 |
|  | CDKExt | XGB | 0.741 | 0.742 | 0.762 | 0.721 | 0.483 | 0.812 |
|  | FP4C | KNN | 0.705 | 0.707 | 0.772 | 0.642 | 0.417 | 0.821 |
|  | FP4C | MLP | 0.501 | 0.503 | 0.597 | 0.409 | 0.007 | 0.891 |
|  | FP4C | PLS | 0.717 | 0.718 | 0.738 | 0.698 | 0.436 | 0.792 |
|  | FP4C | RF | 0.739 | 0.739 | 0.757 | 0.721 | 0.478 | 0.814 |
|  | FP4C | SVM | 0.734 | 0.734 | 0.728 | 0.740 | 0.468 | 0.803 |
|  | FP4C | XGB | 0.760 | 0.760 | 0.767 | 0.753 | 0.520 | 0.811 |
|  | MACCS | KNN | 0.658 | 0.660 | 0.738 | 0.581 | 0.323 | 0.785 |
|  | MACCS | MLP | 0.508 | 0.508 | 0.515 | 0.502 | 0.017 | 0.577 |
|  | MACCS | PLS | 0.637 | 0.637 | 0.636 | 0.637 | 0.273 | 0.709 |
|  | MACCS | RF | 0.703 | 0.703 | 0.704 | 0.702 | 0.406 | 0.798 |
|  | MACCS | SVM | 0.670 | 0.670 | 0.665 | 0.674 | 0.339 | 0.742 |
|  | MACCS | XGB | 0.713 | 0.713 | 0.728 | 0.698 | 0.426 | 0.807 |
|  | Pubchem | KNN | 0.720 | 0.721 | 0.757 | 0.684 | 0.442 | 0.855 |
|  | Pubchem | MLP | 0.734 | 0.734 | 0.748 | 0.721 | 0.468 | 0.812 |
|  | Pubchem | PLS | 0.774 | 0.776 | 0.835 | 0.716 | 0.554 | 0.833 |
|  | Pubchem | RF | 0.781 | 0.782 | 0.786 | 0.777 | 0.563 | 0.849 |
|  | Pubchem | SVM | 0.746 | 0.746 | 0.738 | 0.753 | 0.491 | 0.824 |
|  | Pubchem | XGB | 0.770 | 0.770 | 0.782 | 0.758 | 0.540 | 0.840 |
| BTS2 | AP2DC | KNN | 0.672 | 0.668 | 0.752 | 0.584 | 0.342 | 0.821 |
|  | AP2DC | MLP | 0.725 | 0.725 | 0.728 | 0.721 | 0.449 | 0.798 |
|  | AP2DC | PLS | 0.712 | 0.713 | 0.694 | 0.732 | 0.425 | 0.796 |
|  | AP2DC | RF | 0.742 | 0.743 | 0.718 | 0.768 | 0.487 | 0.845 |
|  | AP2DC | SVM | 0.763 | 0.762 | 0.777 | 0.747 | 0.524 | 0.826 |
|  | AP2DC | XGB | 0.747 | 0.747 | 0.757 | 0.737 | 0.494 | 0.824 |
|  | CDKExt | KNN | 0.710 | 0.705 | 0.811 | 0.600 | 0.421 | 0.830 |
|  | CDKExt | MLP | 0.737 | 0.739 | 0.709 | 0.768 | 0.477 | 0.808 |
|  | CDKExt | PLS | 0.737 | 0.737 | 0.757 | 0.716 | 0.474 | 0.821 |
|  | CDKExt | RF | 0.760 | 0.759 | 0.782 | 0.737 | 0.519 | 0.838 |
|  | CDKExt | SVM | 0.778 | 0.777 | 0.791 | 0.763 | 0.555 | 0.837 |
|  | CDKExt | XGB | 0.740 | 0.739 | 0.757 | 0.721 | 0.479 | 0.810 |
|  | FP4C | KNN | 0.692 | 0.691 | 0.714 | 0.668 | 0.382 | 0.829 |
|  | FP4C | MLP | 0.510 | 0.515 | 0.388 | 0.642 | 0.031 | 0.816 |
|  | FP4C | PLS | 0.717 | 0.717 | 0.718 | 0.716 | 0.434 | 0.796 |
|  | FP4C | RF | 0.755 | 0.754 | 0.772 | 0.737 | 0.509 | 0.836 |
|  | FP4C | SVM | 0.750 | 0.750 | 0.748 | 0.753 | 0.500 | 0.817 |
|  | FP4C | XGB | 0.770 | 0.770 | 0.786 | 0.753 | 0.539 | 0.849 |
|  | MACCS | KNN | 0.697 | 0.695 | 0.743 | 0.647 | 0.392 | 0.794 |
|  | MACCS | MLP | 0.523 | 0.520 | 0.592 | 0.447 | 0.040 | 0.536 |
|  | MACCS | PLS | 0.669 | 0.667 | 0.718 | 0.616 | 0.336 | 0.742 |
|  | MACCS | RF | 0.763 | 0.762 | 0.782 | 0.742 | 0.524 | 0.817 |
|  | MACCS | SVM | 0.755 | 0.755 | 0.757 | 0.753 | 0.510 | 0.803 |
|  | MACCS | XGB | 0.747 | 0.746 | 0.782 | 0.711 | 0.494 | 0.800 |
|  | Pubchem | KNN | 0.689 | 0.689 | 0.689 | 0.689 | 0.379 | 0.836 |
|  | Pubchem | MLP | 0.735 | 0.735 | 0.743 | 0.726 | 0.469 | 0.774 |
|  | Pubchem | PLS | 0.753 | 0.753 | 0.752 | 0.753 | 0.505 | 0.827 |
|  | Pubchem | RF | 0.755 | 0.755 | 0.762 | 0.747 | 0.509 | 0.826 |
|  | Pubchem | SVM | 0.730 | 0.730 | 0.718 | 0.742 | 0.460 | 0.809 |
|  | Pubchem | XGB | 0.717 | 0.715 | 0.772 | 0.658 | 0.433 | 0.802 |
| BTS3 | AP2DC | KNN | 0.722 | 0.721 | 0.786 | 0.655 | 0.446 | 0.858 |
|  | AP2DC | MLP | 0.757 | 0.756 | 0.772 | 0.741 | 0.513 | 0.827 |
|  | AP2DC | PLS | 0.702 | 0.702 | 0.704 | 0.701 | 0.404 | 0.773 |
|  | AP2DC | RF | 0.757 | 0.757 | 0.762 | 0.751 | 0.513 | 0.854 |
|  | AP2DC | SVM | 0.774 | 0.773 | 0.806 | 0.741 | 0.548 | 0.851 |
|  | AP2DC | XGB | 0.767 | 0.767 | 0.767 | 0.766 | 0.533 | 0.831 |
|  | CDKExt | KNN | 0.715 | 0.713 | 0.786 | 0.640 | 0.431 | 0.843 |
|  | CDKExt | MLP | 0.742 | 0.741 | 0.772 | 0.711 | 0.484 | 0.844 |
|  | CDKExt | PLS | 0.732 | 0.732 | 0.743 | 0.721 | 0.464 | 0.820 |
|  | CDKExt | RF | 0.747 | 0.746 | 0.782 | 0.711 | 0.494 | 0.849 |
|  | CDKExt | SVM | 0.744 | 0.744 | 0.777 | 0.711 | 0.489 | 0.827 |
|  | CDKExt | XGB | 0.722 | 0.721 | 0.752 | 0.690 | 0.444 | 0.824 |
|  | FP4C | KNN | 0.725 | 0.723 | 0.791 | 0.655 | 0.451 | 0.850 |
|  | FP4C | MLP | 0.521 | 0.524 | 0.383 | 0.665 | 0.050 | 0.818 |
|  | FP4C | PLS | 0.700 | 0.700 | 0.694 | 0.706 | 0.400 | 0.761 |
|  | FP4C | RF | 0.759 | 0.759 | 0.791 | 0.726 | 0.519 | 0.824 |
|  | FP4C | SVM | 0.774 | 0.774 | 0.796 | 0.751 | 0.548 | 0.832 |
|  | FP4C | XGB | 0.752 | 0.751 | 0.772 | 0.731 | 0.503 | 0.830 |
|  | MACCS | KNN | 0.697 | 0.696 | 0.762 | 0.629 | 0.395 | 0.809 |
|  | MACCS | MLP | 0.476 | 0.475 | 0.524 | 0.426 | -0.050 | 0.446 |
|  | MACCS | PLS | 0.667 | 0.668 | 0.660 | 0.675 | 0.335 | 0.749 |
|  | MACCS | RF | 0.769 | 0.769 | 0.762 | 0.777 | 0.539 | 0.843 |
|  | MACCS | SVM | 0.747 | 0.747 | 0.762 | 0.731 | 0.493 | 0.812 |
|  | MACCS | XGB | 0.757 | 0.756 | 0.791 | 0.721 | 0.514 | 0.848 |
|  | Pubchem | KNN | 0.687 | 0.687 | 0.714 | 0.660 | 0.374 | 0.808 |
|  | Pubchem | MLP | 0.702 | 0.702 | 0.723 | 0.680 | 0.404 | 0.790 |
|  | Pubchem | PLS | 0.722 | 0.722 | 0.733 | 0.711 | 0.444 | 0.820 |
|  | Pubchem | RF | 0.749 | 0.749 | 0.748 | 0.751 | 0.499 | 0.840 |
|  | Pubchem | SVM | 0.725 | 0.725 | 0.694 | 0.756 | 0.451 | 0.800 |
|  | Pubchem | XGB | 0.732 | 0.732 | 0.748 | 0.716 | 0.464 | 0.825 |
| BTS4 | AP2DC | KNN | 0.723 | 0.719 | 0.777 | 0.661 | 0.441 | 0.830 |
|  | AP2DC | MLP | 0.736 | 0.736 | 0.728 | 0.744 | 0.472 | 0.801 |
|  | AP2DC | PLS | 0.699 | 0.700 | 0.699 | 0.700 | 0.398 | 0.799 |
|  | AP2DC | RF | 0.728 | 0.728 | 0.733 | 0.722 | 0.455 | 0.819 |
|  | AP2DC | SVM | 0.731 | 0.730 | 0.743 | 0.717 | 0.459 | 0.824 |
|  | AP2DC | XGB | 0.749 | 0.749 | 0.748 | 0.750 | 0.497 | 0.826 |
|  | CDKExt | KNN | 0.702 | 0.695 | 0.796 | 0.594 | 0.400 | 0.799 |
|  | CDKExt | MLP | 0.723 | 0.721 | 0.743 | 0.700 | 0.443 | 0.771 |
|  | CDKExt | PLS | 0.738 | 0.740 | 0.718 | 0.761 | 0.478 | 0.783 |
|  | CDKExt | RF | 0.762 | 0.761 | 0.772 | 0.750 | 0.522 | 0.819 |
|  | CDKExt | SVM | 0.738 | 0.738 | 0.738 | 0.739 | 0.476 | 0.804 |
|  | CDKExt | XGB | 0.736 | 0.736 | 0.728 | 0.744 | 0.472 | 0.811 |
|  | FP4C | KNN | 0.699 | 0.697 | 0.733 | 0.661 | 0.395 | 0.813 |
|  | FP4C | MLP | 0.479 | 0.492 | 0.306 | 0.678 | -0.018 | 0.789 |
|  | FP4C | PLS | 0.668 | 0.671 | 0.636 | 0.706 | 0.341 | 0.733 |
|  | FP4C | RF | 0.741 | 0.740 | 0.757 | 0.722 | 0.480 | 0.818 |
|  | FP4C | SVM | 0.728 | 0.727 | 0.738 | 0.717 | 0.454 | 0.793 |
|  | FP4C | XGB | 0.749 | 0.747 | 0.772 | 0.722 | 0.495 | 0.805 |
|  | MACCS | KNN | 0.671 | 0.667 | 0.723 | 0.611 | 0.337 | 0.786 |
|  | MACCS | MLP | 0.490 | 0.489 | 0.505 | 0.472 | -0.023 | 0.560 |
|  | MACCS | PLS | 0.632 | 0.634 | 0.607 | 0.661 | 0.267 | 0.674 |
|  | MACCS | RF | 0.720 | 0.721 | 0.714 | 0.728 | 0.440 | 0.791 |
|  | MACCS | SVM | 0.702 | 0.703 | 0.689 | 0.717 | 0.405 | 0.739 |
|  | MACCS | XGB | 0.741 | 0.738 | 0.777 | 0.700 | 0.478 | 0.804 |
|  | Pubchem | KNN | 0.728 | 0.724 | 0.782 | 0.667 | 0.452 | 0.853 |
|  | Pubchem | MLP | 0.775 | 0.771 | 0.820 | 0.722 | 0.546 | 0.842 |
|  | Pubchem | PLS | 0.764 | 0.763 | 0.786 | 0.739 | 0.526 | 0.842 |
|  | Pubchem | RF | 0.764 | 0.765 | 0.757 | 0.772 | 0.528 | 0.824 |
|  | Pubchem | SVM | 0.782 | 0.781 | 0.796 | 0.767 | 0.563 | 0.824 |
|  | Pubchem | XGB | 0.775 | 0.772 | 0.811 | 0.733 | 0.546 | 0.848 |
| BTS5 | AP2DC | KNN | 0.733 | 0.733 | 0.796 | 0.670 | 0.470 | 0.835 |
|  | AP2DC | MLP | 0.753 | 0.753 | 0.791 | 0.714 | 0.507 | 0.814 |
|  | AP2DC | PLS | 0.751 | 0.750 | 0.767 | 0.734 | 0.501 | 0.793 |
|  | AP2DC | RF | 0.756 | 0.756 | 0.718 | 0.793 | 0.513 | 0.839 |
|  | AP2DC | SVM | 0.763 | 0.763 | 0.757 | 0.768 | 0.526 | 0.827 |
|  | AP2DC | XGB | 0.729 | 0.729 | 0.743 | 0.714 | 0.457 | 0.821 |
|  | CDKExt | KNN | 0.743 | 0.743 | 0.767 | 0.719 | 0.487 | 0.835 |
|  | CDKExt | MLP | 0.748 | 0.748 | 0.767 | 0.729 | 0.496 | 0.803 |
|  | CDKExt | PLS | 0.736 | 0.736 | 0.762 | 0.709 | 0.472 | 0.801 |
|  | CDKExt | RF | 0.760 | 0.760 | 0.767 | 0.754 | 0.521 | 0.827 |
|  | CDKExt | SVM | 0.758 | 0.758 | 0.782 | 0.734 | 0.516 | 0.827 |
|  | CDKExt | XGB | 0.758 | 0.758 | 0.786 | 0.729 | 0.516 | 0.808 |
|  | FP4C | KNN | 0.721 | 0.721 | 0.801 | 0.640 | 0.447 | 0.845 |
|  | FP4C | MLP | 0.555 | 0.554 | 0.650 | 0.458 | 0.111 | 0.827 |
|  | FP4C | PLS | 0.726 | 0.726 | 0.733 | 0.719 | 0.452 | 0.800 |
|  | FP4C | RF | 0.773 | 0.772 | 0.791 | 0.754 | 0.545 | 0.842 |
|  | FP4C | SVM | 0.773 | 0.773 | 0.767 | 0.778 | 0.545 | 0.841 |
|  | FP4C | XGB | 0.780 | 0.780 | 0.796 | 0.764 | 0.560 | 0.855 |
|  | MACCS | KNN | 0.704 | 0.704 | 0.752 | 0.655 | 0.410 | 0.826 |
|  | MACCS | MLP | 0.472 | 0.471 | 0.534 | 0.409 | -0.058 | 0.452 |
|  | MACCS | PLS | 0.697 | 0.697 | 0.704 | 0.690 | 0.394 | 0.736 |
|  | MACCS | RF | 0.763 | 0.763 | 0.733 | 0.793 | 0.527 | 0.830 |
|  | MACCS | SVM | 0.721 | 0.721 | 0.714 | 0.729 | 0.443 | 0.785 |
|  | MACCS | XGB | 0.736 | 0.736 | 0.752 | 0.719 | 0.472 | 0.802 |
|  | Pubchem | KNN | 0.719 | 0.718 | 0.786 | 0.650 | 0.441 | 0.842 |
|  | Pubchem | MLP | 0.756 | 0.756 | 0.748 | 0.764 | 0.511 | 0.835 |
|  | Pubchem | PLS | 0.758 | 0.758 | 0.777 | 0.739 | 0.516 | 0.829 |
|  | Pubchem | RF | 0.765 | 0.765 | 0.762 | 0.768 | 0.531 | 0.851 |
|  | Pubchem | SVM | 0.763 | 0.763 | 0.752 | 0.773 | 0.526 | 0.816 |
|  | Pubchem | XGB | 0.753 | 0.753 | 0.752 | 0.754 | 0.506 | 0.829 |

## **Table S6** Independent test results of 30 base-classifiers developed based on individual five training balanced datasets (i.e., BTS1–BTS5).

| **Dataset** | **Descriptor** | **Method** | **ACC** | **BACC** | **SN** | **SP** | **MCC** | **AUC** |
| --- | --- | --- | --- | --- | --- | --- | --- | --- |
| BTS1 | AP2DC | KNN | 0.638 | 0.724 | 0.855 | 0.593 | 0.339 | 0.810 |
|  | AP2DC | MLP | 0.711 | 0.716 | 0.725 | 0.708 | 0.340 | 0.805 |
|  | AP2DC | PLS | 0.688 | 0.754 | 0.855 | 0.653 | 0.388 | 0.800 |
|  | AP2DC | RF | 0.736 | 0.772 | 0.826 | 0.717 | 0.425 | 0.848 |
|  | AP2DC | SVM | 0.716 | 0.719 | 0.725 | 0.714 | 0.346 | 0.806 |
|  | AP2DC | XGB | 0.688 | 0.708 | 0.739 | 0.678 | 0.323 | 0.807 |
|  | CDKExt | KNN | 0.658 | 0.730 | 0.841 | 0.620 | 0.350 | 0.838 |
|  | CDKExt | MLP | 0.643 | 0.733 | 0.870 | 0.596 | 0.352 | 0.827 |
|  | CDKExt | PLS | 0.744 | 0.771 | 0.812 | 0.729 | 0.426 | 0.846 |
|  | CDKExt | RF | 0.698 | 0.743 | 0.812 | 0.675 | 0.374 | 0.859 |
|  | CDKExt | SVM | 0.754 | 0.777 | 0.812 | 0.742 | 0.438 | 0.849 |
|  | CDKExt | XGB | 0.701 | 0.750 | 0.826 | 0.675 | 0.385 | 0.843 |
|  | FP4C | KNN | 0.661 | 0.697 | 0.754 | 0.641 | 0.302 | 0.837 |
|  | FP4C | MLP | 0.173 | 0.500 | 1.000 | 0.000 | 0.000 | 0.902 |
|  | FP4C | PLS | 0.706 | 0.708 | 0.710 | 0.705 | 0.326 | 0.789 |
|  | FP4C | RF | 0.809 | 0.781 | 0.739 | 0.824 | 0.478 | 0.833 |
|  | FP4C | SVM | 0.759 | 0.751 | 0.739 | 0.763 | 0.406 | 0.825 |
|  | FP4C | XGB | 0.746 | 0.715 | 0.667 | 0.763 | 0.351 | 0.810 |
|  | MACCS | KNN | 0.628 | 0.701 | 0.812 | 0.590 | 0.304 | 0.812 |
|  | MACCS | MLP | 0.804 | 0.509 | 0.058 | 0.960 | 0.035 | 0.695 |
|  | MACCS | PLS | 0.608 | 0.671 | 0.768 | 0.574 | 0.259 | 0.745 |
|  | MACCS | RF | 0.749 | 0.762 | 0.783 | 0.742 | 0.416 | 0.837 |
|  | MACCS | SVM | 0.681 | 0.698 | 0.725 | 0.672 | 0.307 | 0.780 |
|  | MACCS | XGB | 0.754 | 0.731 | 0.696 | 0.766 | 0.376 | 0.838 |
|  | Pubchem | KNN | 0.666 | 0.758 | 0.899 | 0.617 | 0.391 | 0.908 |
|  | Pubchem | MLP | 0.714 | 0.781 | 0.884 | 0.678 | 0.431 | 0.879 |
|  | Pubchem | PLS | 0.696 | 0.770 | 0.884 | 0.657 | 0.413 | 0.836 |
|  | Pubchem | RF | 0.774 | 0.823 | 0.899 | 0.748 | 0.508 | 0.883 |
|  | Pubchem | SVM | 0.744 | 0.805 | 0.899 | 0.711 | 0.472 | 0.877 |
|  | Pubchem | XGB | 0.714 | 0.775 | 0.870 | 0.681 | 0.423 | 0.841 |
| BTS2 | AP2DC | KNN | 0.653 | 0.704 | 0.783 | 0.626 | 0.311 | 0.809 |
|  | AP2DC | MLP | 0.683 | 0.740 | 0.826 | 0.653 | 0.367 | 0.807 |
|  | AP2DC | PLS | 0.741 | 0.746 | 0.754 | 0.739 | 0.392 | 0.844 |
|  | AP2DC | RF | 0.771 | 0.776 | 0.783 | 0.769 | 0.445 | 0.845 |
|  | AP2DC | SVM | 0.779 | 0.803 | 0.841 | 0.766 | 0.485 | 0.852 |
|  | AP2DC | XGB | 0.764 | 0.783 | 0.812 | 0.754 | 0.450 | 0.853 |
|  | CDKExt | KNN | 0.603 | 0.703 | 0.855 | 0.550 | 0.307 | 0.825 |
|  | CDKExt | MLP | 0.701 | 0.745 | 0.812 | 0.678 | 0.377 | 0.812 |
|  | CDKExt | PLS | 0.691 | 0.727 | 0.783 | 0.672 | 0.350 | 0.807 |
|  | CDKExt | RF | 0.724 | 0.764 | 0.826 | 0.702 | 0.410 | 0.856 |
|  | CDKExt | SVM | 0.704 | 0.758 | 0.841 | 0.675 | 0.396 | 0.832 |
|  | CDKExt | XGB | 0.726 | 0.766 | 0.826 | 0.705 | 0.413 | 0.853 |
|  | FP4C | KNN | 0.716 | 0.714 | 0.710 | 0.717 | 0.338 | 0.834 |
|  | FP4C | MLP | 0.173 | 0.500 | 1.000 | 0.000 | 0.000 | 0.855 |
|  | FP4C | PLS | 0.739 | 0.722 | 0.696 | 0.748 | 0.357 | 0.793 |
|  | FP4C | RF | 0.807 | 0.786 | 0.754 | 0.818 | 0.481 | 0.827 |
|  | FP4C | SVM | 0.769 | 0.757 | 0.739 | 0.775 | 0.419 | 0.826 |
|  | FP4C | XGB | 0.771 | 0.770 | 0.768 | 0.772 | 0.438 | 0.830 |
|  | MACCS | KNN | 0.643 | 0.698 | 0.783 | 0.614 | 0.302 | 0.824 |
|  | MACCS | MLP | 0.827 | 0.500 | 1.000 | 0.000 | 0.000 | 0.410 |
|  | MACCS | PLS | 0.641 | 0.691 | 0.768 | 0.614 | 0.291 | 0.769 |
|  | MACCS | RF | 0.726 | 0.731 | 0.739 | 0.723 | 0.366 | 0.823 |
|  | MACCS | SVM | 0.724 | 0.770 | 0.841 | 0.699 | 0.418 | 0.822 |
|  | MACCS | XGB | 0.759 | 0.785 | 0.826 | 0.745 | 0.452 | 0.821 |
|  | Pubchem | KNN | 0.698 | 0.772 | 0.884 | 0.660 | 0.415 | 0.875 |
|  | Pubchem | MLP | 0.774 | 0.749 | 0.710 | 0.787 | 0.411 | 0.801 |
|  | Pubchem | PLS | 0.741 | 0.763 | 0.797 | 0.729 | 0.415 | 0.840 |
|  | Pubchem | RF | 0.746 | 0.761 | 0.783 | 0.739 | 0.413 | 0.836 |
|  | Pubchem | SVM | 0.754 | 0.777 | 0.812 | 0.742 | 0.438 | 0.843 |
|  | Pubchem | XGB | 0.769 | 0.791 | 0.826 | 0.757 | 0.464 | 0.838 |
| BTS3 | AP2DC | KNN | 0.646 | 0.706 | 0.797 | 0.614 | 0.312 | 0.833 |
|  | AP2DC | MLP | 0.746 | 0.715 | 0.667 | 0.763 | 0.351 | 0.775 |
|  | AP2DC | PLS | 0.751 | 0.781 | 0.826 | 0.736 | 0.442 | 0.847 |
|  | AP2DC | RF | 0.761 | 0.753 | 0.739 | 0.766 | 0.409 | 0.842 |
|  | AP2DC | SVM | 0.726 | 0.708 | 0.681 | 0.736 | 0.334 | 0.741 |
|  | AP2DC | XGB | 0.731 | 0.746 | 0.768 | 0.723 | 0.387 | 0.799 |
|  | CDKExt | KNN | 0.671 | 0.738 | 0.841 | 0.635 | 0.362 | 0.856 |
|  | CDKExt | MLP | 0.688 | 0.743 | 0.826 | 0.660 | 0.372 | 0.797 |
|  | CDKExt | PLS | 0.673 | 0.739 | 0.841 | 0.638 | 0.365 | 0.803 |
|  | CDKExt | RF | 0.706 | 0.759 | 0.841 | 0.678 | 0.399 | 0.852 |
|  | CDKExt | SVM | 0.688 | 0.743 | 0.826 | 0.660 | 0.372 | 0.799 |
|  | CDKExt | XGB | 0.729 | 0.761 | 0.812 | 0.711 | 0.408 | 0.848 |
|  | FP4C | KNN | 0.678 | 0.742 | 0.841 | 0.644 | 0.370 | 0.863 |
|  | FP4C | MLP | 0.827 | 0.500 | 1.000 | 0.000 | 0.000 | 0.796 |
|  | FP4C | PLS | 0.759 | 0.757 | 0.754 | 0.760 | 0.414 | 0.804 |
|  | FP4C | RF | 0.774 | 0.760 | 0.739 | 0.781 | 0.426 | 0.812 |
|  | FP4C | SVM | 0.779 | 0.757 | 0.725 | 0.790 | 0.426 | 0.836 |
|  | FP4C | XGB | 0.764 | 0.737 | 0.696 | 0.778 | 0.390 | 0.818 |
|  | MACCS | KNN | 0.606 | 0.698 | 0.841 | 0.556 | 0.301 | 0.821 |
|  | MACCS | MLP | 0.239 | 0.540 | 1.000 | 0.079 | 0.121 | 0.495 |
|  | MACCS | PLS | 0.653 | 0.710 | 0.797 | 0.623 | 0.320 | 0.767 |
|  | MACCS | RF | 0.704 | 0.752 | 0.826 | 0.678 | 0.388 | 0.823 |
|  | MACCS | SVM | 0.698 | 0.760 | 0.855 | 0.666 | 0.399 | 0.833 |
|  | MACCS | XGB | 0.721 | 0.717 | 0.710 | 0.723 | 0.344 | 0.807 |
|  | Pubchem | KNN | 0.709 | 0.766 | 0.855 | 0.678 | 0.409 | 0.864 |
|  | Pubchem | MLP | 0.729 | 0.802 | 0.913 | 0.690 | 0.463 | 0.856 |
|  | Pubchem | PLS | 0.731 | 0.780 | 0.855 | 0.705 | 0.434 | 0.881 |
|  | Pubchem | RF | 0.761 | 0.833 | 0.942 | 0.723 | 0.516 | 0.888 |
|  | Pubchem | SVM | 0.749 | 0.802 | 0.884 | 0.720 | 0.470 | 0.865 |
|  | Pubchem | XGB | 0.756 | 0.807 | 0.884 | 0.729 | 0.479 | 0.868 |
| BTS4 | AP2DC | KNN | 0.643 | 0.733 | 0.870 | 0.596 | 0.352 | 0.830 |
|  | AP2DC | MLP | 0.784 | 0.726 | 0.638 | 0.815 | 0.389 | 0.811 |
|  | AP2DC | PLS | 0.744 | 0.759 | 0.783 | 0.736 | 0.410 | 0.814 |
|  | AP2DC | RF | 0.749 | 0.745 | 0.739 | 0.751 | 0.393 | 0.829 |
|  | AP2DC | SVM | 0.784 | 0.766 | 0.739 | 0.793 | 0.440 | 0.830 |
|  | AP2DC | XGB | 0.771 | 0.753 | 0.725 | 0.781 | 0.415 | 0.849 |
|  | CDKExt | KNN | 0.643 | 0.727 | 0.855 | 0.599 | 0.344 | 0.851 |
|  | CDKExt | MLP | 0.729 | 0.727 | 0.725 | 0.729 | 0.361 | 0.825 |
|  | CDKExt | PLS | 0.683 | 0.746 | 0.841 | 0.650 | 0.375 | 0.825 |
|  | CDKExt | RF | 0.736 | 0.777 | 0.841 | 0.714 | 0.432 | 0.858 |
|  | CDKExt | SVM | 0.726 | 0.783 | 0.870 | 0.696 | 0.437 | 0.863 |
|  | CDKExt | XGB | 0.726 | 0.748 | 0.783 | 0.714 | 0.389 | 0.834 |
|  | FP4C | KNN | 0.656 | 0.729 | 0.841 | 0.617 | 0.347 | 0.859 |
|  | FP4C | MLP | 0.173 | 0.500 | 1.000 | 0.000 | 0.000 | 0.970 |
|  | FP4C | PLS | 0.759 | 0.751 | 0.739 | 0.763 | 0.406 | 0.807 |
|  | FP4C | RF | 0.786 | 0.751 | 0.696 | 0.805 | 0.422 | 0.809 |
|  | FP4C | SVM | 0.756 | 0.750 | 0.739 | 0.760 | 0.403 | 0.816 |
|  | FP4C | XGB | 0.756 | 0.778 | 0.812 | 0.745 | 0.441 | 0.831 |
|  | MACCS | KNN | 0.661 | 0.703 | 0.768 | 0.638 | 0.311 | 0.822 |
|  | MACCS | MLP | 0.827 | 0.500 | 1.000 | 0.000 | 0.000 | 0.955 |
|  | MACCS | PLS | 0.696 | 0.707 | 0.725 | 0.690 | 0.323 | 0.779 |
|  | MACCS | RF | 0.751 | 0.752 | 0.754 | 0.751 | 0.404 | 0.836 |
|  | MACCS | SVM | 0.734 | 0.724 | 0.710 | 0.739 | 0.359 | 0.795 |
|  | MACCS | XGB | 0.731 | 0.711 | 0.681 | 0.742 | 0.340 | 0.808 |
|  | Pubchem | KNN | 0.746 | 0.789 | 0.855 | 0.723 | 0.452 | 0.918 |
|  | Pubchem | MLP | 0.741 | 0.821 | 0.942 | 0.699 | 0.493 | 0.875 |
|  | Pubchem | PLS | 0.749 | 0.808 | 0.899 | 0.717 | 0.478 | 0.850 |
|  | Pubchem | RF | 0.764 | 0.817 | 0.899 | 0.736 | 0.496 | 0.883 |
|  | Pubchem | SVM | 0.774 | 0.835 | 0.928 | 0.742 | 0.523 | 0.880 |
|  | Pubchem | XGB | 0.749 | 0.808 | 0.899 | 0.717 | 0.478 | 0.865 |
| BTS5 | AP2DC | KNN | 0.668 | 0.713 | 0.783 | 0.644 | 0.327 | 0.815 |
|  | AP2DC | MLP | 0.711 | 0.728 | 0.754 | 0.702 | 0.356 | 0.808 |
|  | AP2DC | PLS | 0.711 | 0.739 | 0.783 | 0.696 | 0.372 | 0.825 |
|  | AP2DC | RF | 0.746 | 0.766 | 0.797 | 0.736 | 0.421 | 0.858 |
|  | AP2DC | SVM | 0.741 | 0.752 | 0.768 | 0.736 | 0.399 | 0.828 |
|  | AP2DC | XGB | 0.759 | 0.780 | 0.812 | 0.748 | 0.444 | 0.852 |
|  | CDKExt | KNN | 0.613 | 0.697 | 0.826 | 0.568 | 0.299 | 0.794 |
|  | CDKExt | MLP | 0.623 | 0.732 | 0.899 | 0.565 | 0.351 | 0.782 |
|  | CDKExt | PLS | 0.663 | 0.716 | 0.797 | 0.635 | 0.330 | 0.796 |
|  | CDKExt | RF | 0.686 | 0.741 | 0.826 | 0.657 | 0.369 | 0.865 |
|  | CDKExt | SVM | 0.714 | 0.781 | 0.884 | 0.678 | 0.431 | 0.851 |
|  | CDKExt | XGB | 0.719 | 0.767 | 0.841 | 0.693 | 0.412 | 0.843 |
|  | FP4C | KNN | 0.716 | 0.737 | 0.768 | 0.705 | 0.370 | 0.874 |
|  | FP4C | MLP | 0.173 | 0.500 | 1.000 | 0.000 | 0.000 | 0.780 |
|  | FP4C | PLS | 0.714 | 0.706 | 0.696 | 0.717 | 0.327 | 0.778 |
|  | FP4C | RF | 0.739 | 0.745 | 0.754 | 0.736 | 0.388 | 0.808 |
|  | FP4C | SVM | 0.766 | 0.750 | 0.725 | 0.775 | 0.408 | 0.815 |
|  | FP4C | XGB | 0.744 | 0.742 | 0.739 | 0.745 | 0.387 | 0.819 |
|  | MACCS | KNN | 0.613 | 0.680 | 0.783 | 0.578 | 0.273 | 0.801 |
|  | MACCS | MLP | 0.201 | 0.517 | 1.000 | 0.033 | 0.077 | 0.716 |
|  | MACCS | PLS | 0.621 | 0.667 | 0.739 | 0.596 | 0.254 | 0.745 |
|  | MACCS | RF | 0.759 | 0.757 | 0.754 | 0.760 | 0.414 | 0.838 |
|  | MACCS | SVM | 0.726 | 0.783 | 0.870 | 0.696 | 0.437 | 0.835 |
|  | MACCS | XGB | 0.724 | 0.747 | 0.783 | 0.711 | 0.386 | 0.817 |
|  | Pubchem | KNN | 0.688 | 0.760 | 0.870 | 0.650 | 0.397 | 0.883 |
|  | Pubchem | MLP | 0.744 | 0.782 | 0.841 | 0.723 | 0.441 | 0.879 |
|  | Pubchem | PLS | 0.731 | 0.769 | 0.826 | 0.711 | 0.419 | 0.844 |
|  | Pubchem | RF | 0.749 | 0.796 | 0.870 | 0.723 | 0.463 | 0.862 |
|  | Pubchem | SVM | 0.764 | 0.800 | 0.855 | 0.745 | 0.473 | 0.857 |
|  | Pubchem | XGB | 0.726 | 0.771 | 0.841 | 0.702 | 0.421 | 0.846 |

## **Table S7** Independent test MCC values of 30 base-classifiers developed based on individual five balanced training datasets.

| **Descriptor** | **Method** | **BTS1** | **BTS2** | **BTS3** | **BTS4** | **BTS5** | **Average** |
| --- | --- | --- | --- | --- | --- | --- | --- |
| AP2DC | KNN | 0.339 | 0.311 | 0.312 | 0.352 | 0.327 | 0.328 |
|  | MLP | 0.340 | 0.367 | 0.351 | 0.389 | 0.356 | 0.360 |
|  | PLS | 0.388 | 0.392 | 0.442 | 0.410 | 0.372 | 0.401 |
|  | RF | 0.425 | 0.445 | 0.409 | 0.393 | 0.421 | 0.419 |
|  | SVM | 0.346 | 0.485 | 0.334 | 0.440 | 0.399 | 0.401 |
|  | XGB | 0.323 | 0.450 | 0.387 | 0.415 | 0.444 | 0.404 |
| CDKExt | KNN | 0.350 | 0.307 | 0.362 | 0.344 | 0.299 | 0.332 |
|  | MLP | 0.352 | 0.377 | 0.372 | 0.361 | 0.351 | 0.363 |
|  | PLS | 0.426 | 0.350 | 0.365 | 0.375 | 0.330 | 0.369 |
|  | RF | 0.374 | 0.410 | 0.399 | 0.432 | 0.369 | 0.397 |
|  | SVM | 0.438 | 0.396 | 0.372 | 0.437 | 0.431 | 0.415 |
|  | XGB | 0.385 | 0.413 | 0.408 | 0.389 | 0.412 | 0.402 |
| FP4C | KNN | 0.302 | 0.338 | 0.370 | 0.347 | 0.370 | 0.345 |
|  | MLP | 0.000 | 0.000 | 0.000 | 0.000 | 0.000 | 0.000 |
|  | PLS | 0.326 | 0.357 | 0.414 | 0.406 | 0.327 | 0.366 |
|  | RF | 0.478 | 0.481 | 0.426 | 0.422 | 0.388 | 0.439 |
|  | SVM | 0.406 | 0.419 | 0.426 | 0.403 | 0.408 | 0.413 |
|  | XGB | 0.351 | 0.438 | 0.390 | 0.441 | 0.387 | 0.401 |
| MACCS | KNN | 0.304 | 0.302 | 0.301 | 0.311 | 0.273 | 0.298 |
|  | MLP | 0.035 | 0.000 | 0.121 | 0.000 | 0.077 | 0.047 |
|  | PLS | 0.259 | 0.291 | 0.320 | 0.323 | 0.254 | 0.289 |
|  | RF | 0.416 | 0.366 | 0.388 | 0.404 | 0.414 | 0.397 |
|  | SVM | 0.307 | 0.418 | 0.399 | 0.359 | 0.437 | 0.384 |
|  | XGB | 0.376 | 0.452 | 0.344 | 0.340 | 0.386 | 0.380 |
| Pubchem | KNN | 0.391 | 0.415 | 0.409 | 0.452 | 0.397 | 0.413 |
|  | MLP | 0.431 | 0.411 | 0.463 | 0.493 | 0.441 | 0.448 |
|  | PLS | 0.413 | 0.415 | 0.434 | 0.478 | 0.419 | 0.432 |
|  | RF | 0.508 | 0.413 | 0.516 | 0.496 | 0.463 | 0.479 |
|  | SVM | 0.472 | 0.438 | 0.470 | 0.523 | 0.473 | 0.475 |
|  | XGB | 0.423 | 0.464 | 0.479 | 0.478 | 0.421 | 0.453 |

## **Table S8** Comparative performance among top-five base-classifiers trained with the imbalanced and balanced datasets over the ten-fold cross-validation test.

| **Dataset** | **Method** | **ACC** | **BACC** | **SN** | **SP** | **MCC** | **AUC** |
| --- | --- | --- | --- | --- | --- | --- | --- |
| Imbalanced | SVM-CDKExt | 0.751 | 0.772 | 0.806 | 0.739 | 0.431 | 0.841 |
|  | RF-CDKExt | 0.741 | 0.771 | 0.816 | 0.726 | 0.425 | 0.851 |
|  | RF-Pubchem | 0.767 | 0.761 | 0.752 | 0.771 | 0.424 | 0.834 |
|  | XGB-FP4C | 0.751 | 0.763 | 0.782 | 0.744 | 0.418 | 0.828 |
|  | XGB-Pubchem | 0.743 | 0.762 | 0.791 | 0.733 | 0.414 | 0.837 |
| Balanced | RF-Pubchem_SS1 | 0.781 | 0.782 | 0.786 | 0.777 | 0.563 | 0.849 |
|  | SVM-Pubchem_SS4 | 0.782 | 0.781 | 0.796 | 0.767 | 0.563 | 0.824 |
|  | XGB-FP4C_SS5 | 0.780 | 0.780 | 0.796 | 0.764 | 0.560 | 0.855 |
|  | SVM-CDKExt_SS2 | 0.778 | 0.777 | 0.791 | 0.763 | 0.555 | 0.837 |
|  | PLS-Pubchem_SS1 | 0.774 | 0.776 | 0.835 | 0.716 | 0.554 | 0.833 |

## **Table S9** Comparative performance among top-five base-classifiers trained with the imbalanced and balanced datasets over the independent test.

| **Dataset** | **Method** | **ACC** | **BACC** | **SN** | **SP** | **MCC** | **AUC** |
| --- | --- | --- | --- | --- | --- | --- | --- |
| Imbalanced | SVM-CDKExt | 0.709 | 0.772 | 0.870 | 0.675 | 0.418 | 0.857 |
|  | RF-CDKExt | 0.721 | 0.774 | 0.855 | 0.693 | 0.423 | 0.852 |
|  | RF-Pubchem | 0.734 | 0.776 | 0.841 | 0.711 | 0.429 | 0.867 |
|  | XGB-FP4C | 0.726 | 0.766 | 0.826 | 0.705 | 0.413 | 0.840 |
|  | XGB-Pubchem | 0.769 | 0.791 | 0.826 | 0.757 | 0.464 | 0.836 |
| Balanced | RF-Pubchem_SS1 | 0.774 | 0.823 | 0.899 | 0.748 | 0.508 | 0.883 |
|  | SVM-Pubchem_SS4 | 0.774 | 0.835 | 0.928 | 0.742 | 0.523 | 0.880 |
|  | XGB-FP4C_SS5 | 0.744 | 0.742 | 0.739 | 0.745 | 0.387 | 0.819 |
|  | SVM-CDKExt_SS2 | 0.704 | 0.758 | 0.841 | 0.675 | 0.396 | 0.832 |
|  | PLS-Pubchem_SS1 | 0.696 | 0.770 | 0.884 | 0.657 | 0.413 | 0.836 |

## **Table S10** Comparative performance among different feature subsets containing *m* top-ranked important PFs in terms of BACC, MCC, and AUC over the cross-validation and independent test.

| **Number of top-ranked features** | **Cross-validation test** | | | **Independent test** | | |
| --- | --- | --- | --- | --- | --- | --- |
|  | **BACC** | **MCC** | **AUC** | **BACC** | **MCC** | **AUC** |
| 10 | 0.860 | 0.625 | 0.932 | 0.874 | 0.622 | 0.932 |
| 20 | 0.860 | 0.621 | 0.940 | 0.856 | 0.576 | 0.940 |
| 30 | 0.875 | 0.652 | 0.945 | 0.887 | 0.639 | 0.945 |
| 40 | 0.878 | 0.653 | 0.949 | 0.890 | 0.638 | 0.949 |
| 50 | 0.874 | 0.644 | 0.947 | 0.886 | 0.635 | 0.947 |
| 60 | 0.875 | 0.645 | 0.946 | 0.877 | 0.649 | 0.946 |
| 70 | 0.880 | 0.655 | 0.950 | 0.881 | 0.623 | 0.950 |
| 80 | 0.875 | 0.648 | 0.949 | 0.868 | 0.622 | 0.949 |
| 90 | 0.890 | 0.679 | 0.953 | 0.886 | 0.635 | 0.953 |
| 100 | 0.901 | 0.693 | 0.963 | 0.873 | 0.618 | 0.963 |
| 110 | 0.885 | 0.668 | 0.962 | 0.904 | 0.686 | 0.962 |
| 120 | 0.890 | 0.689 | 0.959 | 0.896 | 0.644 | 0.959 |
| 130 | 0.888 | 0.673 | 0.959 | 0.877 | 0.631 | 0.959 |
| 140 | 0.900 | 0.708 | 0.964 | 0.891 | 0.658 | 0.953 |
| 150 | 0.903 | 0.713 | 0.965 | 0.882 | 0.643 | 0.944 |

## **Table S11** List of the top 20 compounds from FDA-approved drugs with their probability score, rank, compound name, docking scores and indicated use.

| **Drug Bank**  **ID** | | **Probability score** | **Rank** | | | **Compound**  **Name** | **Docking score (kcal/mol)** | | **Indicated use** | |
| --- | --- | --- | --- | --- | --- | --- | --- | --- | --- | --- |
| DB00834 | | 0.9985 | | | 3 | Mifepristone | | -11.4 | Medical abortion and early miscarriage | |
| DB08867 | | 0.9991 | | | 2 | Ulipristal | | -10.7 | Emergency contraception  and uterine fibroids | |
| DB00717 | | 0.9976 | | | 6 | Norethisterone | | -9.5 | Contraception, and endometriosis | |
| DB12474 | | 0.9965 | | | 25 | Lynestrenol | | -9.5 | Contraception, and gynecological disorders | |
| DB00972 | | 0.9966 | | | 24 | Azelastine | | -9.3 | Allergic rhinitis, or other upper respiratory allergies | |
| DB00294 | | 0.9966 | | | 23 | Etonogestrel | | -9.1 | Contraception under the skin of the upper arm | |
| DB06713 | | 0.9962 | | | 29 | Norelgestromin | | -9.1 | Contraception in combination with an estrogen | |
| DB09070 | | 0.9967 | | | 22 | Tibolone | | -8.9 | Hormone replacement therapy containing a synthetic hormone | |
| DB13520 | | 0.9980 | | | 5 | Metergoline | | -8.8 | Inhibition of prolactin | |
| DB00367 | | 0.9974 | | | 10 | Levonorgestrel | | -8.8 | Emergency contraceptive | |
| DB09389 | | 0.9974 | | | 11 | Norgestrel | | -8.8 | Oral contraception | |
| DB09371 | | 0.9968 | | | 19 | Norethynodrel | | -8.8 | Functional uterine bleeding and endometriosis | |
| DB00624 | | 0.9963 | | | 28 | Testosterone | | -8.8 | Hypogonadism, breast carcinoma and symptoms of menopause | |
| DB08883 | | 0.9984 | | | 4 | Perampanel | | -8.7 | The first drug approved for epilepsy | |
| DB11901 | | 0.9992 | | | 1 | Apalutamide | | -8.6 | Non-metastatic castration-resistant prostate cancer | |
| DB00990 | | 0.9967 | | | 21 | Exemestane | | -8.6 | Breast cancer in postmenopausal women after treatment with tamoxifen | |
| DB06710 | | 0.9976 | | | 7 | Methyltestosterone | | -8.4 | Replacement therapy in conditions associated with testosterone deficiencies in males | |
| DB09123 | | 0.9974 | | | 9 | Dienogest | | -8.4 | Endometriosis or contraception in combination with ethinylestradiol | |
| DB00670 | | 0.9965 | | | 26 | Pirenzepine | | -8.3 | Peptic ulcers, gastric ulcers, and duodenal ulcers | |
| DB1213 | | 0.9961 | | | 30 | Lorlatinib | | -8.0 | Anaplastic lymphoma kinase positive metastatic non-small cell lung cancer | |

## **Table S12.** ∆*G*_bind_ values (kcal/mol) and energetic components of drugs in complex with GR calculated by the MM/GB(PB)SA methods

|  | **MF** | | **AZE** | | **PER** | | **MET** | | **PIR** | |
| --- | --- | --- | --- | --- | --- | --- | --- | --- | --- | --- |
|  | **MM/GBSA** | **MM/PBSA** | **MM/GBSA** | **MM/PBSA** | **MM/GBSA** | **MM/PBSA** | **MM/GBSA** | **MM/PBSA** | **MM/GBSA** | **MM/PBSA** |
| **∆E_ele_** | −15.7 ± 0.4 | −15.7 ± 0.3 | −6.0 ± 0.3 | −6.0 ± 0.2 | −14.8 ± 0.4 | −14.8 ± 0.4 | −4.4 ± 0.3 | −4.5 ± 0.3 | −8.5 ± 0.3 | −8.5 ± 0.3 |
| **∆E_vdW_** | −65.0 ± 0.3 | −65.0 ± 0.3 | −54.9 ± 0.3 | −54.9 ± 0.3 | −54.3 ± 0.3 | −54.3 ± 0.3 | −51.1 ± 0.3 | −51.1 ± 0.3 | −44.0 ± 0.3 | −44.0 ± 0.3 |
| **∆E_MM_** | −80.7 ± 0.7 | −80.7 ± 0.6 | −60.9 ± 0.6 | −60.9 ± 0.5 | −69.1 ± 0.7 | −69.1 ± 0.7 | −55.5 ± 0.6 | −55.6 ± 0.6 | −52.5 ± 0.6 | −52.5 ± 0.6 |
| **∆G_nonpolar,sol_** | −7.6 ± 0.1 | −7.7 ± 0.1 | −6.2 ± 0.1 | −6.4 ± 0.1 | −6.8 ± 0.1 | −6.4 ± 0.1 | −6.3 ± 0.1 | −7.2 ± 0.1 | −5.5 ± 0.1 | −5.7 ± 0.1 |
| **∆G_ele,sol_** | 36.2 ± 0.3 | 43.6 ± 0.4 | 20.0 ± 0.2 | 26.5 ± 0.3 | 30.1 ± 0.3 | 36.6 ± 0.3 | 17.9 ± 0.3 | 25.8 ± 0.3 | 24.8 ± 0.2 | 29.2 ± 0.3 |
| **∆G_sol_** | 28.6 ± 0.3 | 35.5 ± 0.5 | 13.8 ± 0.2 | 20.1 ± 0.2 | 23.3 ± 0.3 | 30.2 ± 0.3 | 11.6 ± 0.3 | 18.6 ± 0.3 | 19.2 ± 0.2 | 23.4 ± 0.3 |
| **∆G_ele,sol_ + ∆E_ele_** | 20.5 ± 0.7 | 19.8 ± 0.7 | 14.0 ± 0.5 | 20.5 ± 0.5 | 15.3 ± 0.7 | 21.8 ± 0.7 | 13.5 ± 0.6 | 21.3 ± 0.6 | 16.3 ± 0.5 | 20.7 ± 0.6 |
| **∆G_nonpolar,sol_ + ∆E_vdW_** | −72.6 ± 0.4 | −72.7 ± 0.4 | −61.1 ± 0.4 | −61.3 ± 0.4 | −61.1 ± 0.4 | −60.7 ± 0.4 | −57.4 ± 0.4 | −58.3 ± 0.4 | −49.5 ± 0.4 | −49.7 ± 0.4 |
| **−T∆S** | −26.4 ± 0.8 | −26.4 ± 0.8 | −20.6 ± 0.4 | −20.6 ± 0.4 | −21.8 ± 0.7 | −21.8 ± 0.7 | −23.3 ± 0.9 | −23.3 ± 0.9 | −22.1 ± 0.8 | −22.1 ± 0.8 |
| **∆G_bind_** | −25.6 ± 1.0 | −18.5 ± 1.0 | −26.5 ± 1.2 | −20.2 ± 1.1 | −23.9 ± 1.2 | −17.0 ± 1.2 | −20.7 ± 1.2 | −13.8 ± 1.2 | −11.2 ± 1.4 | −7.0 ± 1.4 |
